# Virally-induced expression of GABA_A_ receptor δ subunits following their pathological loss reveals their role in regulating GABA_A_ receptor assembly

**DOI:** 10.1016/j.pneurobio.2022.102337

**Published:** 2022-08-05

**Authors:** Yu Sun, Zechun Peng, Xiaofei Wei, Nianhui Zhang, Christine S. Huang, Martin Wallner, Istvan Mody, Carolyn R. Houser

**Affiliations:** a Department of Neurobiology, USA; b Department of Molecular and Medical Pharmacology, USA; c Departmentof Neurology, USA; d Brain Research Institute, David Geffen School of Medicine at the University of California, Los Angeles, Los Angeles, CA 90095, USA

**Keywords:** Delta subunit, Dentate granule cells, Epilepsy, GABA_A_ receptors, Neurosteroids, Tonic inhibition

## Abstract

Decreased expression of the δ subunit of the GABA_A_ receptor (GABA_A_R) has been found in the dentate gyrus in several animal models of epilepsy and other disorders with increased excitability and is associated with altered modulation of tonic inhibition in dentate granule cells (GCs). In contrast, other GABA_A_R subunits, including α4 and γ2 subunits, are increased, but the relationship between these changes is unclear. The goals of this study were to determine if viral transfection of δ subunits in dentate GCs could increase δ subunit expression, alter expression of potentially-related GABA_A_R subunits, and restore more normal network excitability in the dentate gyrus in a mouse model of epilepsy. Pilocarpine-induced seizures were elicited in DOCK10-Cre mice that express Cre selectively in dentate GCs, and two weeks later the mice were injected unilaterally with a Cre-dependent δ-GABA_A_R viral vector. At 4–6 weeks following transfection, δ subunit immunolabeling was substantially increased in dentate GCs on the transfected side compared to the nontransfected side. Importantly, α4 and γ2 subunit labeling was downregulated on the transfected side. Electrophysiological studies revealed enhanced tonic inhibition, decreased network excitability, and increased neurosteroid sensitivity in slices from the δ subunit-transfected side compared to those from the nontransfected side of the same pilocarpine-treated animal, consistent with the formation of δ subunit-containing GABA_A_Rs. No differences were observed between sides of eYFP-transfected animals. These findings are consistent with the idea that altering expression of key subunits, such as the δ subunit, regulates GABA_A_R subunit assemblies, resulting in substantial effects on network excitability.

## 1. Introduction

GABA_A_ receptors (GABA_A_Rs) are major mediators of inhibition throughout the central nervous system (CNS) and, as such, are altered in numerous disease processes that involve increased excitability (Braat and Kooy, 2015; Lee and Maguire, 2014; Möhler, 2006; Whissell et al., 2015, for reviews). These alterations are particularly challenging to address because of the wide diversity of GABA_A_Rs, due, in part, to the number of different subunits (currently nineteen) that can assemble to form heteropentameric GABA-gated chloride channels (Olsen and Sieghart, 2008). The native GABA_A_Rs are generally composed of two α subunits, two β subunits, and either a γ or δ subunit that assemble in a number of preferred combinations. The specific subunit composition of the GABA_A_Rs is critical as it determines their pharmacological characteristics, channel properties, subcellular localization, and associated function, including their role as mediators of phasic or tonic inhibition (Belelli et al., 2009; Farrant and Nusser, 2005; Fritschy and Panzanelli, 2014; Hannan et al., 2020; Macdonald and Olsen, 1994; Sieghart and Sperk, 2002, for reviews).

Much has been learned about the subunit composition, formation and function of GABA_A_R subtypes from in vitro studies of recombinant receptors in various cell lines following transfection for multiple GABA_A_R subunits which, in many cases, are not normally expressed by the cells. Much less is known about responses to similar manipulations in mature neurons in vivo in which multiple GABA_A_R receptors already exist but may have been altered by disease processes. Despite the inherent complexity of in vivo systems, such basic information could serve as a framework for the development of new disease-modifying treatments for both genetic and acquired disorders with altered excitability, potentially focusing on alterations in specific subunits.

A number of GABA_A_R subunits are altered in animal models of epilepsy (Brooks-Kayal et al., 1998; Fritschy, 2008; Gonzalez et al., 2015; Houser and Esclapez, 2003; Peng et al., 2004; Rajasekaran et al., 2010; Schwarzer et al., 1997) as well as humans with temporal lobe and neocortical epilepsy (Joshi et al., 2020; Loup et al., 2000; Pirker et al., 2003). These subunit changes are complex and often include both increases and decreases in GABA_A_R subunits. The functional effects of the multiple changes have been difficult to determine but will depend on the identity of the subunits that are decreased as well as the subunit composition and function of GABA_A_Rs that may be formed (Coulter, 2001; Olsen and Sieghart, 2009).

The increased expression of several subunits, including γ2, α4, and β2/3, has generally been viewed as a compensatory response to control excitability whereas decreased expression of subunits such as α1 and δ subunits has been considered potentially contributory (Fritschy, 2008; Houser et al., 2012; Sperk et al., 2004, for reviews). This has led to a search for ways to selectively increase the expression or enhance the function of the deficient GABA_A_R subunits (Braat and Kooy, 2015; Chuang and Reddy, 2018; Raol et al., 2006; Yu et al., 2019). The δ subunit was selected for the current study as decreases in δ subunit expression and function have been identified in a number of animal models with increased excitability. These include models of status epilepticus-induced epilepsy (Peng et al., 2004; Rajasekaran et al., 2010; Schwarzer et al., 1997); some genetic models of epilepsy (Lee et al., 2021; Payne et al., 2006); and models of traumatic brain injury (Boychuk et al., 2016; Parga Becerra et al., 2021), alcohol withdrawal (Cagetti et al., 2003), fragile X syndrome (Curia et al., 2009; Sabanov et al., 2017; Zhang et al., 2017), and postpartum depression (Maguire and Mody, 2008). Thus the current study has implications beyond a single model of epilepsy and addresses basic questions of GABA_A_R plasticity and function in vivo.

A decrease in the δ subunit is of particular interest because δ subunit-containing GABA_A_Rs are major mediators of tonic inhibition in the dentate gyrus (Brickley and Mody, 2012; Glykys et al., 2008) and are located at nonsynaptic sites where they are ideally positioned to respond to ambient levels of GABA or spillover of GABA at highly active synapses (Nusser et al., 1998; Wei et al., 2003). Consistent with their nonsynaptic or perisynaptic locations, δ subunit-containing GABA_A_Rs have a high affinity for GABA and slow rates of desensitization (Glykys and Mody, 2007a; Haas and Macdonald, 1999; Lagrange et al., 2018). Importantly, δ subunit-containing receptors are also highly sensitive to modulation by neurosteroids and low levels of ethanol (Belelli et al., 2009; Mihalek et al., 1999; Stell et al., 2003; Wallner et al., 2003; Wohlfarth et al., 2002). Considering these characteristics, a decrease in δ subunit expression could contribute to increased excitability in the dentate gyrus and leave the region without sufficient modulatory control of tonic inhibition, which could be particularly detrimental during seizure-promoting conditions.

Interestingly, the α4 subunit, a major partner of the δ subunit in the dentate gyrus, is increased rather than decreased in several models of epilepsy, and the γ2 subunit is also frequently increased in the same models (Peng et al., 2004; Rajasekaran et al., 2010; Schwarzer et al., 1997). Such changes have led to suggestions that new GABA_A_Rs with altered subunit composition could be formed in epilepsy and other disorders with increased excitability (Cagetti et al., 2003; Joshi and Kapur, 2019; Olsen and Spigelman, 2012). Specifically, in several models, α4βγ2 GABA_A_Rs appeared to be compensating for the lack of extrasynaptic α4βδ receptors. While α4βγ2 GABA_A_Rs could potentially compensate for the decrease in δ subunit-containing receptors by increasing phasic inhibition or maintaining tonic inhibition, they would lack key features of the normally predominant δ subunit-containing receptors (α4βδ). Previous studies of recombinant receptors in mouse cell lines have demonstrated by direct comparisons that α4βγ2 receptors are much less sensitive to GABA than α4βδ receptors and show decreased modulation by neuroactive steroids, including tetrahydrodeox-ycorticosterone (THDOC) (Brown et al., 2002). Likewise, in those animal models of epilepsy and traumatic brain injury in which δ subunits are decreased but tonic inhibition is maintained, the responsible receptors, presumably α4βγ2, continue to show decreased neurosteroid sensitivity (Boychuk et al., 2016; Payne et al., 2006; Rajasekaran et al., 2010; Zhang et al., 2007).

Such findings suggest that restoring more normal, modifiable tonic inhibition would require not only an increase in δ subunit expression but also assembly of the newly synthesized subunit in GABA_A_Rs that could be trafficked to the cell surface and inserted at nonsynaptic sites in the plasma membrane. The process would likely require changes in multiple GABA_A_R subunits and the establishment of functionally appropriate subunit partnerships.

Three GABA_A_R subunits (δ, α4 and γ2) were of special interest as alterations in these subunits often occur together in the same brain region in a number of animal models. It is unclear whether the changes occur independently during the disease process or whether the decrease in the δ subunit could be a critical precipitating event that could lead to increased expression of the α4 and γ2 subunits. To address this question, we used an adeno-associated virus (AAV) vector to increase Crerecombinase (Cre)-dependent δ subunit expression selectively in dentate granule cells (GCs) and determine the functional effects of this transfection, including effects on expression of the α4 and γ2 subunits.

The specific goals were to determine whether 1) Cre-dependent viral transfection for the δ subunit in dentate GCs would increase δ subunit expression in a model in which the epileptogenic process was ongoing; 2) the δ subunit would be trafficked to the cell surface, as necessary for functional GABA_A_Rs; 3) changes in subunit expression would be restricted to the δ subunit or whether the α4 and γ2 subunits would also be modified; and 4) a virally-induced increase in δ subunit expression would lead to functional GABA_A_Rs that would decrease excitability of dentate GCs and increase their responsiveness to modulation by the neurosteroid THDOC. Preliminary reports of some of these findings have been presented previously in abstract form (Peng et al., 2019).

## 2. Material and methods

### 2.1. Animals and experimental design

The DOCK10-Cre mice used in this study express Cre specifically in dentate GCs. Production of these mice, also referred to as Dentate Gyrus Granule Cell (DGGC) specific-Cre mice, has been described in detail by Kohara et al. (2014), and mice for establishing the DOCK10-Cre colony were generously provided by Susumu Tonegawa. The mice were produced and maintained on a C57BL/6 background.

**Fig. 1.**
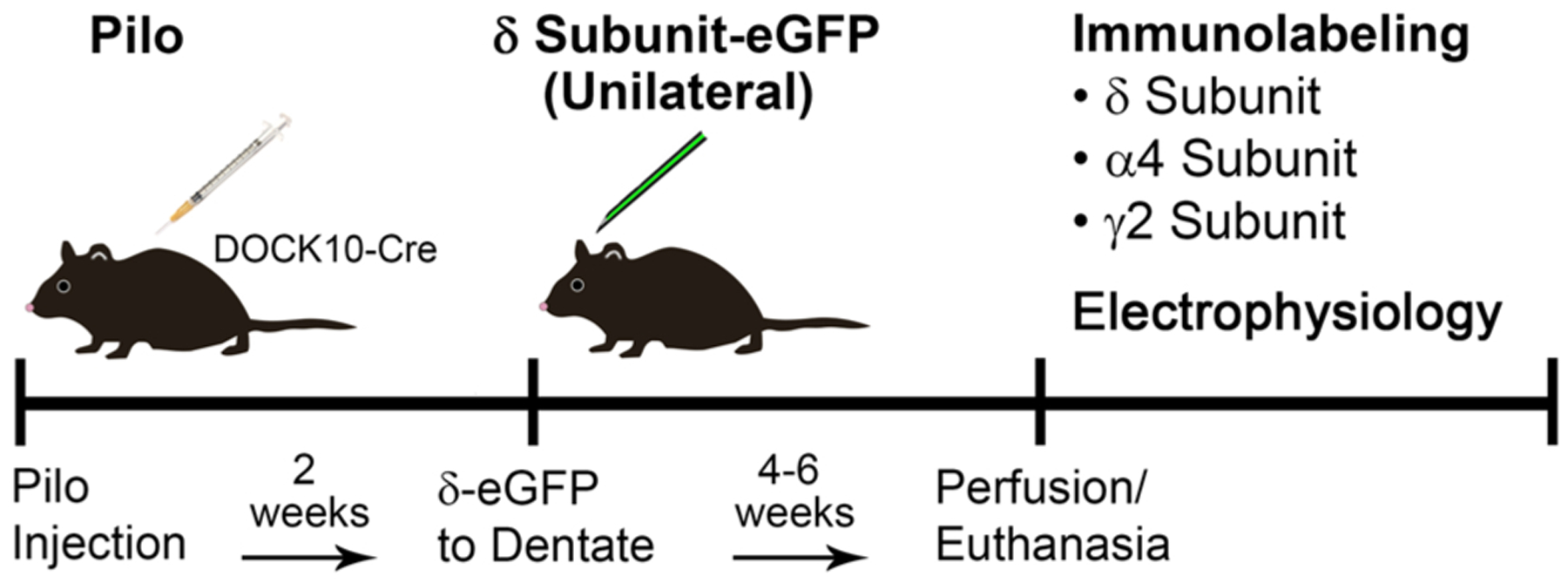
Timecourse of the experimental procedures. DOCK10-Cre mice were treated with pilocarpine (pilo) to induce status epilepticus and create a mouse model of epilepsy. Two weeks later, the mice were transfected unilaterally with an AAV vector containing a Cre-inducible δ subunit-eGFP (or control eYFP) construct. After 4–6 weeks, the effects of the transfection were determined by comparing immunolabeling for GABA_A_R subunits and electrophysiological responses on the transfected and nontransfected sides.

At 6–7 weeks of age, DOCK10-Cre animals were treated with pilocarpine to elicit a period of status epilepticus (Fig. 1). At two weeks post pilocarpine treatment, a subgroup of the animals was transfected unilaterally with a Cre-dependent viral construct for the δ subunit fused with eGFP (enhanced green fluorescent protein) (Fig. 1). A second group of animals was transfected unilaterally with Cre-dependent eYFP (control for δ transfection) at the same timepoint. A third group of pilocarpine-treated mice received no transfection. A final group of mice received saline rather than pilocarpine injections and had no transfection (control for pilocarpine). At 4–6 weeks after transfection, the animals were either perfused for immunohistochemical studies of GABA_A_R subunit localization or used for electrophysiological studies of network excitability and neurosteroid sensitivity.

The study was designed to determine the effects of introducing the δ subunit following, rather than preceding, the initial episode of status epilepticus. Likewise, if considering use for treatment, an early post-insult timepoint could be most effective. A two-week post-pilocarpine timepoint was selected after pilot studies with transfections performed at one week led to increased cell loss in the hippocampus, including dentate GCs. The two-week interval allowed time for recovery from the status epilepticus episode and yet was relatively early in the post-insult period. The eYFP and δ subunit-eGFP transfections were made *unilaterally* in separate animals. We specifically opted for this experimental design to ensure within-animal controls for variations in immunohistochemical labeling and electrophysiological recordings that were not related to the transfections.

Male mice were used in this study as δ subunit levels have been found to fluctuate during the estrus cycle (Maguire et al., 2005), and it is unknown how such changes could affect expression of the δ subunit following viral transfection of the subunit and the related functional studies that are the focus of the current study.

The numbers of DOCK10 mice (n = 36) varied among the groups and included: n = 21 for light microscopy (7 controls with no pilocarpine; 5 pilocarpine-treated with no transfection; 7 pilocarpine-treated with unilateral δ subunit transfection; 2 pilocarpine-treated with unilateral eYFP transfection); n = 3 for electron microscopy (1 control with no pilocarpine; 2 pilocarpine-treated with unilateral δ subunit transfection); n = 12 for electrophysiology (2 controls with no pilocarpine; 3 pilocarpine-treated with no transfection; 4 pilocarpine-treated with unilateral δ subunit transfection; 3 pilocarpine-treated with unilateral eYFP transfection). All animal-use protocols conformed to the National Institutes of Health guidelines and were approved by the University of California, Los Angeles, Chancellor’s Animal Research Committee.

### 2.2. Pilocarpine treatment

The pilocarpine mouse model of recurrent seizures has been described in detail in previous studies (Peng et al., 2004, 2013). Briefly, a single episode of seizure activity (status epilepticus) is induced; the animals progressively recover over the next 1–2 weeks (acute period); and they then begin exhibiting spontaneous behavioral seizures, characteristic of epilepsy, with these recurrent seizures continuing through the remainder of life (chronic period).

In the current study, young adult (6–8 weeks) male DOCK10-Cre mice (C57BL/6 background) were used, and status epilepticus was induced in experimental animals by the administration of pilocarpine, a muscarinic cholinergic agonist. Mice were initially injected with scopolamine methyl nitrate (1 mg/kg, s.c.; Sigma-Aldrich) to reduce peripheral cholinergic effects. Thirty min later, experimental animals in each group received an injection of pilocarpine hydrochloride (300–340 mg/kg, s.c.; Sigma-Aldrich) to induce status epilepticus. At 2 h after the onset of sustained seizures, diazepam (9.0 mg/kg, s.c.; Hospira) was administered to suppress or ameliorate the acute behavioral seizures. Limiting the duration of status epilepticus contributes to more rapid recovery and more consistent patterns of cell loss, with selective cell loss primarily in the hilus and CA3, but preservation of dentate GCs (Peng and Houser, 2005). Control animals received an identical series of injections except that pilocarpine was replaced with a similar volume of sterile saline. Following the induced seizure episode, pilocarpine-treated animals recovered, generally resumed eating and drinking by the following day, and displayed normal behavior over the next few days. At 2 weeks following pilocarpine treatment, a subgroup of the animals received unilateral transfection of an adeno-associated viral (AAV) vector containing a Cre-dependent δ-eGFP subunit construct or control eYFP construct in the dentate gyrus (Fig. 1), with the goal of increasing δ subunit expression selectively in dentate GCs.

### 2.3. Plasmid construction/characterization and AAV vector production

An AAV vector encoding the Cre-dependent GABA_A_R δ subunit was constructed in order to drive expression of the δ subunit in Cre-expressing neurons. Plasmid construction and characterization have been described in detail previously (Tong et al., 2015). Briefly a C-terminal δ subunit-eGFP fusion construct was made by overlap extension (Horton et al., 1989), and PCR products were cloned into a “double--floxed” Cre-inducible AAV vector in antisense orientation with respect to the ubiquitous EF1α promotor. The δ subunit-eGFP linker sequence coding for GGRARDPPVAT was inserted in-frame between the last C-terminal amino acid of the GABA_A_R δ (rat) clone and the start codon of eGFP. The Cre-inducible AAV vector (pAAV-EF1a-double floxed-hChR2 (H134R)-EYFP-WPRE-HGHpA; Plasmid #20298) was obtained from Addgene; the insert (ChR2-eYFP) was removed by cutting with restriction enzymes *Nhe*I (5’ end) and *Asc*I (3’ end) and replaced with the *Nhe*I *Asc*I cut PCR product coding for the GABA_A_R subunit δ-eGFP fusion construct to obtain the Cre-inducible δ-eGFP construct.

For functional testing, the inducible δ-eGFP fusion construct was co-transfected into HEK293T cells together with a plasmid coding for Cre (pAAV-EF1α-mCherry-IRES-Cre; Addgene Plasmid #55632), and successful Cre-dependent induction was demonstrated by fluorescence microscopy. For electrophysiological tests, HEK293T cells were transfected with the Cre-inducible δ-eGFP and plasmids coding for GABA_A_R subunits α6 and β3, to allow for the formation of functional GABA_A_R subtypes in HEK cells. Receptors with the Cre-inducible δ-eGFP construct produced robust GABA-evoked currents, as described previously (Tong et al., 2015).

The Cre-inducible δ-eGFP construct was packaged into an AAV vector (AAV-DJ-DIO Gabrd-eGFP) by the Stanford University Gene Vector and Virus Core. The AAV-DJ serotype is a chimera of three serotypes (AAV2, 8 and 9) and has been shown to be very efficient for both in vivo and in vitro transductions (Grimm et al., 2008; Large et al., 2021). The DJ serotype is used extensively by the Stanford Core, and a comparable control virus (AAV-DJ-EF1α-DIO eYFP), containing a construct for eYFP, was also obtained from the Stanford Neuroscience Gene Vector and Virus Core.

### 2.4. Viral vector injections

To selectively label dentate GCs, DOCK10-Cre mice were injected with AAV vectors containing the Cre-dependent constructs for either the δ-eGFP subunit or eYFP. For all transfections, injections were made unilaterally in the right dentate gyrus. Mice were anesthetized with isoflurane, and the viral vector was stereotaxically injected in the dentate gyrus with a Nanoject II injector (Drummond Scientific), using glass pipettes. A small animal stereotaxic instrument with digital display console (Model 940; Kopf Instruments) was used for positioning of the pipette in the hilus where the AAV could transfect GCs in both the supraand infrapyramidal blades of the dentate gyrus. For neuroanatomical and electrophysiological studies, injections were made at two sites in close proximity to each other in the rostral dentate gyrus (−1.8, −2.3 mm anteroposterior (AP); 1.0, 1.3 mm mediolateral (ML); 2.2, 2.4 mm dorsoventral (DV)); and at one site, at three depths, in the caudal dentate gyrus (−3.6 mm AP; 2.8 mm ML; 3.0, 3.7, 4.4 mm DV, in relation to Bregma) (Paxinos and Franklin, 2019). Injection volumes were 69–92 nl at each site (23 nl increments × 3–4 injections). Following the injections, the pipette was left in position for 5 min before it was slowly retracted from the brain. Mice were studied at 4–6 weeks post-transfection.

### 2.5. Tissue preparation for light microscopy

All mice used for neuroanatomical studies were deeply anesthetized with Fatal-Plus (90 mg/kg, i.p.) and perfused intracardially with 4% paraformaldehyde in 0.12 M phosphate buffer, pH 7.3. After 1 h at 4 °C, brains were removed and postfixed for 1 h, rinsed, and cryoprotected in a 30% sucrose solution overnight. They were then embedded in OCT compound (Sakura Finetek), frozen on dry ice, and sectioned at 30 μm with a cryostat (CM 3050 S, Leica Microsystems). Brains were sectioned coronally through the rostral half and then horizontally through the caudal half of the hippocampal formation.

### 2.6. Immunohistochemistry for light microscopy

The following GABA_A_R subunit-specific antisera were used in the immunohistochemical studies: rabbit anti-δ (Millipore AB9752, 1:1000); rabbit anti-α4, N-terminus (Millipore AB5457, 1:1000); and rabbit anti-γ2 (319 366), 1:1000, kindly provided by Werner Sieghart (Tretter et al., 1997). Specificity of the δ and α4 antisera was confirmed by a lack of immunolabeling in tissue from the respective knockout animals (Peng et al., 2002, 2014). Specificity of the affinity-purified γ2 antisera has been demonstrated previously (Peng et al., 2002; Sperk et al., 1997). Prior to immunohistochemistry, free-floating sections were incubated in 1% H_2_O_2_ for 30 min and then processed with a water bath heating antigen-retrieval method to reduce endogenous peroxidase-like activity and enhance specific labeling of the receptor subunits (Peng et al., 2002). Briefly, the sections were heated to 90 °C for 70 min in sodium citrate solution (pH 8.6). After cooling and rinsing in 0.1 M Tris buffered saline (TBS, pH 7.3), sections were processed for immunohistochemistry with standard avidin-biotin-peroxidase methods (Vectastain Elite ABC; Vector Laboratories) as described in detail previously (Peng et al., 2002, 2004).

### 2.7. Densitometric analysis

Densitometry was used to compare the intensity of immunolabeling for α4 and γ2 subunits in the molecular layer of the dentate gyrus on the two sides of the same animal in pilocarpine-treated animals with unilateral δ transfection, pilocarpine-treated animals with no transfection, and control animals. For the transfected animals, sections were first processed for δ subunit labeling, and only animals with δ subunit transfection throughout at least 75% of the molecular layer in the same section were included in the densitometric analyses. Sections adjacent to those with δ subunit transfection were processed for α4 and γ2 subunit labeling, with sections from all animals in the group processed identically in the same immunolabeling experiment. Sections at comparable levels in the nontransfected pilocarpine-treated and control groups were selected for α4 and γ2 subunit labeling.

The intensity of immunolabeling was analyzed with an Axioskop 2 microscope equipped with an AxioCam digital camera system and AxioVision 4.6 software (Carl Zeiss). Linear black and white digital images of α4 or γ2 immunolabeling in the dentate molecular layer from each side were obtained under identical conditions on the same day with stabilized light levels. The entire molecular layer of the dentate gyrus was outlined in each image, and the densities of labeling (gray values) within this region were determined with morphometric AxioVision software. All values were corrected for background labeling in the same section. Data from the two sides of each animal were analyzed with a Wilcoxon paired non-parametric test (two-tailed), using Igor Pro 8.04 software (WaveMetrics), and p < 0.05 was considered statistically significant. The percentage differences in intensity of labeling between the two sides were also determined. Subsequently, a Spearman Rank-order Coefficient was used to determine if there was a correlation between the percentage changes in δ subunit labeling and those of α4 and γ2 subunits.

### 2.8. Tissue preparation for electron microscopy

Tissue from a normal DOCK10-Cre mouse and two pilocarpine-treated mice with unilateral δ-eGFP subunit transfection was prepared for postembedding immunogold labeling for the δ subunit, as described previously (Zhang et al., 2007). Following perfusion of the mice with 4% paraformaldehyde and 0.1% glutaraldehyde and removal of the brain, coronal sections of the forebrain that included the hippocampus were cut at 200 μm on a vibratome (VT1000S; Leica Microsystems), and small blocks of the dentate gyrus were trimmed from these sections. These specimens were cryoprotected, frozen at −190 °C in a cryofixation unit (EM CPC; Leica Microsystems), and then transferred to a cryosubstitution unit (EM AFS; Leica) which was programmed for all steps. Specimens were subsequently infiltrated with Lowicryl HM20 resin (Electron Microscopy Sciences), and the resin was polymerized as described previously.

### 2.9. Postembedding immunogold labeling and quantitative analysis

Ultrathin sections were cut on a microtome (Reichert-Jung), picked up on nickel mesh grids, and processed for immunogold labeling with previously described methods (Zhang et al., 2007). Sections from the transfected and nontransfected sides were processed together. After appropriate pre-treatments, sections were incubated in primary antiserum, rabbit anti-δ subunit (Millipore AB9752 or PhosphoSolutions 868-GDN, 1:100) in 0.01 M TBS (pH 7.4) containing 2% human serum albumin (HSA) for 18–24 h at room temperature. Sections were then incubated for 2.5 h in secondary antisera, goat anti-rabbit IgG (H+L) or F(ab’)_2_ fragment of goat-anti-rabbit IgG (Aurion; distributed by Electron Microscopy Sciences) conjugated to 10 nm colloidal gold particles, diluted 1:25 in 0.05 M Tris-HCL, pH 8.0, containing 2% HSA. After immunogold processing, sections were stained with a saturated solution of uranyl acetate in distilled water for 40 min and lead citrate for 4 min

Series of randomly selected fields in the molecular layer of the dentate gyrus containing synaptic profiles were photographed with a JEOL 100CX II electron microscope at a primary magnification of 19,000x. The localization of colloidal gold particles was determined for each symmetric synapse in the photomicrographs. For analysis of δ subunit labeling on transfected and nontransfected sides, synapses were classified as labeled (immunogold particles at perisynaptic or nearby extrasynaptic sites) or unlabeled (no immunogold labeling near the synapse). For further analysis of δ subunit localization, the distribution of gold particles along the postsynaptic membranes was classified as either perisynaptic, extrasynaptic, or synaptic locations. Labeling was operationally defined as perisynaptic if the gold particles were located either directly at the ends of the synaptic contact or within 30 nm of the ends of the synapse. Labeling was defined as extrasynaptic if the gold particles were located up to 100 nm beyond the end of the synapse. Gold particles that were located at extrasynaptic sites farther than 100 nm from the ends of a synaptic contact were not included in this analysis. Labeling was classified as synaptic if gold particles were located directly at synaptic contacts, excluding the perisynaptic sites indicated above. The percentages of δ subunit-labeled and unlabeled synapses and the percentages of immunogold particles at perisynaptic, extrasynaptic and synaptic sites were determined. For the overall analysis, synapses were considered labeled if gold particles were identified at any of these sites. No statistical analyses were used due to the limited number of animals. Regions of the granule cell layer were also photographed, and immunogold labeling within the granule cell cytoplasm was studied and compared qualitatively on the two sides.

### 2.10. Preparation of brain slices for electrophysiology

Mice were anesthetized with isoflurane and decapitated according to a protocol approved by the University of California, Los Angeles, Chancellor’s Animal Research Committee. The brains were quickly removed and coronal 350 μm thick slices were cut on a Leica VT1200S Vibratome in ice-cold N-Methyl-D-Glutamine (NMDG)-based HEPES-buffered solution, containing in mM: 135 NMDG, 10 D-glucose, 4 MgCl_2_, 0.5 CaCl_2_, 1 KCl, 1.2 KH_2_PO_4_, 26 HEPES, bubbled with 100% O_2_, pH 7.4, 290 −300 mOsm. A small part of the left corner of each section was cut off to distinguish the left side from right. All slices were incubated at 32 °C in an interface chamber in a reduced sodium artificial CSF (ACSF), containing in mM: 85 NaCl, 25 D-glucose, 55 sucrose, 2.5 KCl, 1.25 NaH_2_PO_4_, 0.5 CaCl_2_, 4 MgCl_2_, 26 NaHCO_3_, pH 7.3–7.4 when bubbled with 95% O_2_, 5% CO_2_. After 20 min the ACSF was gradually substituted for normal ACSF at 32 °C, containing in mM: 126 NaCl, 10 D-glucose, 2 MgCl_2_, 2 CaCl_2_, 2.5 KCl, 1.25 NaH_2_PO_4_, 1.5 Na Pyruvate, 1 L-Glutamine, 26 NaHCO_3_, pH 7.3–7.4 when bubbled with 95% O2, 5% CO_2_. All salts were purchased from Sigma-Aldrich. After 1 hr recovery, approximately an equal number of slices were transferred either to an interface recording chamber for field potential recordings or to a submerged recording chamber for patch clamp recordings. Both chambers were maintained at 34 °C and perfused at 2 ml/min in the interface chamber and 5 ml/min in the submerged chamber with ACSF containing 5 μM GABA.

### 2.11. Recordings of evoked field EPSPs in the dentate gyrus

Field excitatory postsynaptic potentials (fEPSP) were recorded in the molecular layer or the granule cell layer of the dentate gyrus with borosilicate pipettes (2–4 MΩ) containing normal ACSF and were evoked (paired pulses 50 ms apart every 30 s) by stimulating the perforant path near the hippocampal fissure. Bipolar electrodes delivered a constant current stimulus (A365, World Precision Instruments). At a stimulus width (W) of 60 μs the intensity was adjusted until a threshold response (~0.3 mV in amplitude) was obtained that was recorded over a 10 min stable baseline. The W was then varied (PG4000, Neurodata Instruments) to create stimulus-response curves by delivering two stimulation trials (10 stimuli each), with W ranging from 20 to 240 μs (in 20 and 40 μs increments). After the control trial, THDOC (20 nM; Tocris) was perfused for 10 min before generating a second pair of stimulus-response curves. Data were filtered between 0.10 Hz and 3 KHz, and an in-house data analysis package (EVAN, version 1.3.9) was used to fit a straight line to the initial rising phase of the fEPSP and to measure its amplitude at its peak. The amplitude was then used to represent the magnitude of the fEPSP and plotted against the stimulus charge (Q) to obtain stimulus–response curves. To allow for correct comparisons between experiments, the Q (in pC) was calculated by multiplying the stimulus W by the constant current value of the stimulation. The Q values were binned at approximately every 50 and 100 pC, and the average values of the amplitudes from all the experiments in one category were plotted against the average Q values for the binned intervals. The SD values of fEPSP amplitudes and of the Q were also plotted to indicate the variance in the x and y planes. Next, for a given experiment, the stimulus–response curves of the average values were fit to the Hill equation of the form f(Q) = MAX/[1 + (Q50/Q)^k], where Q is stimulus charge in pC, MAX is the asymptote at + ∞ of the maximum response, k is the slope factor, and Q50 is the charge that elicits 50% of MAX (Igor Pro 8.04, Wavemetrics).

### 2.12. Patch clamp recordings and analysis of tonic currents

Cells were visualized under IR-DIC upright microscopy (Olympus BX-51WI, 20x XLUMPlan FL N objective), and whole-cell recordings under voltage clamp condition were performed on dentate gyrus granule cells with borosilicate patch pipettes (4–6 MΩ, King Precision Glass) containing internal solutions (ICS) (in mM): 140 Cs-methylsulphonate, 2 MgCl_2_, 10 HEPES, 0.2 EGTA, 2 Na_2_-ATP, 0.2 Na_2_-GTP. The pH of the ICS was adjusted to 7.2 with CsOH and its osmolarity was 285–290 mOsm. ICS were stored at − 80 °C in 1 ml aliquots. Before each experiment, ICS aliquots were thawed to room temperature and kept on ice during the experiment. Recordings were obtained using an Axopatch 200B amplifier (Molecular Devices), low-pass filtered at 5 kHz (Bessel, 8-pole) and digitized at 10 kHz with a National Instruments data acquisition board (BNC 2110). All data were acquired with EVAN (custom-designed LabView-based software). The holding potential of whole cell patch clamp recording was set to 0 mV, at the reversal potential of the excitatory glutamatergic currents, to record only the inhibitory post synaptic currents. Series resistance and whole-cell capacitance were estimated from fast transients evoked by a 5-mV voltage command step using lag values of 7 μs and then compensated to 70–80%. Recordings were discontinued if series resistance increased by more than 25% through an experiment or the compensated resistance surpassed 25 MΩ at any time during the experiment. Drugs were perfused after a stable control recording period of at least 2 mins. Only one cell was recorded per slice, therefore, slices experienced no repeated drug perfusions. To measure and calculate tonic current, a custom written procedure (IGOR Pro 8, Wavemetrics) was used. An All-points histogram of a recording segment of 30 s during the period of interest was plotted (Glykys and Mody, 2007b). A Gaussian was fitted to the part of the distribution from the minimum value at the left to the rightmost (largest) value of the histogram distribution. The mean of the fitted Gaussian was considered to be the holding current (*I*_*h*_). This process was repeated for all 30 s epochs of interest, including during the end of perfusion of 50 nM THDOC. The 40 μM gabazine (GBZ) blocked GABA_A_R mediated phasic and tonic currents, and the tonic current of all other recording periods was calculated by *I*_*h*_-*I*_*h*_ in GBZ. The area of the histogram falling outside the Gaussian was taken as the phasic current (Glykys and Mody, 2007b).

### 2.13. Experimental design and statistical analyses

A within-animal experimental design was used for both immunohistochemical and electrophysiological studies. This experimental design involved transfection of either δ subunit-eGFP or eYFP on one side while leaving the other side unaffected. The nontransfected side served as a within-animal control for any changes in immunohistochemical labeling or electrophysiological recordings that were not linked to the unilateral AAV transfection. This design provided controls for numerous variables among pilocarpine-treated animals, including the severity of the initial period of status epilepticus and related histological changes and the occurrence of spontaneous seizures or other behavioral activity that could influence GABA_A_R subunit expression. The within-animal design also allowed for virtually identical immunohistochemical processing of tissue from the two sides (both sides included in the same forebrain section during all processing) and for electrophysiological recordings with the hippocampus from each side in the same recording chamber and perfused with identical reagents, including THDOC.

Differences between the two sides were determined for all conditions. All densitometry data were analyzed with a paired sample Wilcoxon signed rank two-tailed test with significance set to p < 0.05. Electrophysiological data were analyzed with either Wilcoxon signed rank two-tailed test for paired data or Mann-Whitney two-tailed test for unpaired data with significance set to p < 0.05. In field potential recordings, whenever possible (>90% of the experiments), two slices, one from each side (L and R), were incubated and recorded from simultaneously. For these recordings, the data were collected and analyzed by an investigator blinded as to the origin of the slices, and experiments were unblinded only after all analyses were completed. For statistical comparisons we found the MAX values obtained by Hill equation fits and representing the asymptotes of the curves at + ∞ to be impractical, as such large stimuli could never be delivered to the slices. Therefore, for statistical comparisons (Wilcoxon signed rank test with significance level p < 0.05), we considered the MAX values to be the averages of the 9–12 fEPSP amplitudes (representing ~10% of all responses) evoked by the largest stimuli delivered to all the slices in a given group. Differences in the fitted Q50 parameters were determined by calculating the 95% and 99% confidence intervals (CI) of the fitted parameters as follows: CI = Q50 ± ZxSD/SQRT(n) where Q50 is the value of the parameter from the fit to the Hill equation, Z is the value from the Z distribution table (1.960 for 95% and 2.576 for 99%), SD is the standard deviation of the fitted Q50, and n is the number of slices. Once the CI were established, if no overlaps were present between the 95% CI or the 99% CI, the Q50 values were considered to be significantly different at the p < 0.05 or p < 0.01 levels, respectively. To establish the effects of 20 nM THDOC on the stimulus-response curves, for each separate experiment, the fEPSP amplitudes evoked by the largest stimulus before THDOC perfusion were considered to have a value of 1.0. The largest fEPSP amplitudes recorded in the presence of THDOC were expressed as relative to this value, and statistical comparisons for determining the significance of the THDOC effects were also done using the responses evoked at the highest stimuli (n = 3 animals per group). The data were fitted to a large number of points (e.g., Fig. 9), and we compared the fitted Q50 (equivalent to EC50 in dose/response curves) and Hill coefficients using a standard t-test. The t-values and the p-values were calculated using the SEMs obtained from the coefficients derived by the fits, thus considering the variance of the data points fitted (GraphPad Prism). This is analogous to the statistical comparison of two dose/response curves.

## 3. Results

### 3.1. Selective Cre-dependent labeling and previously described GABA_A_R subunit changes were confirmed in DOCK10-Cre mice

**Fig. 2.**
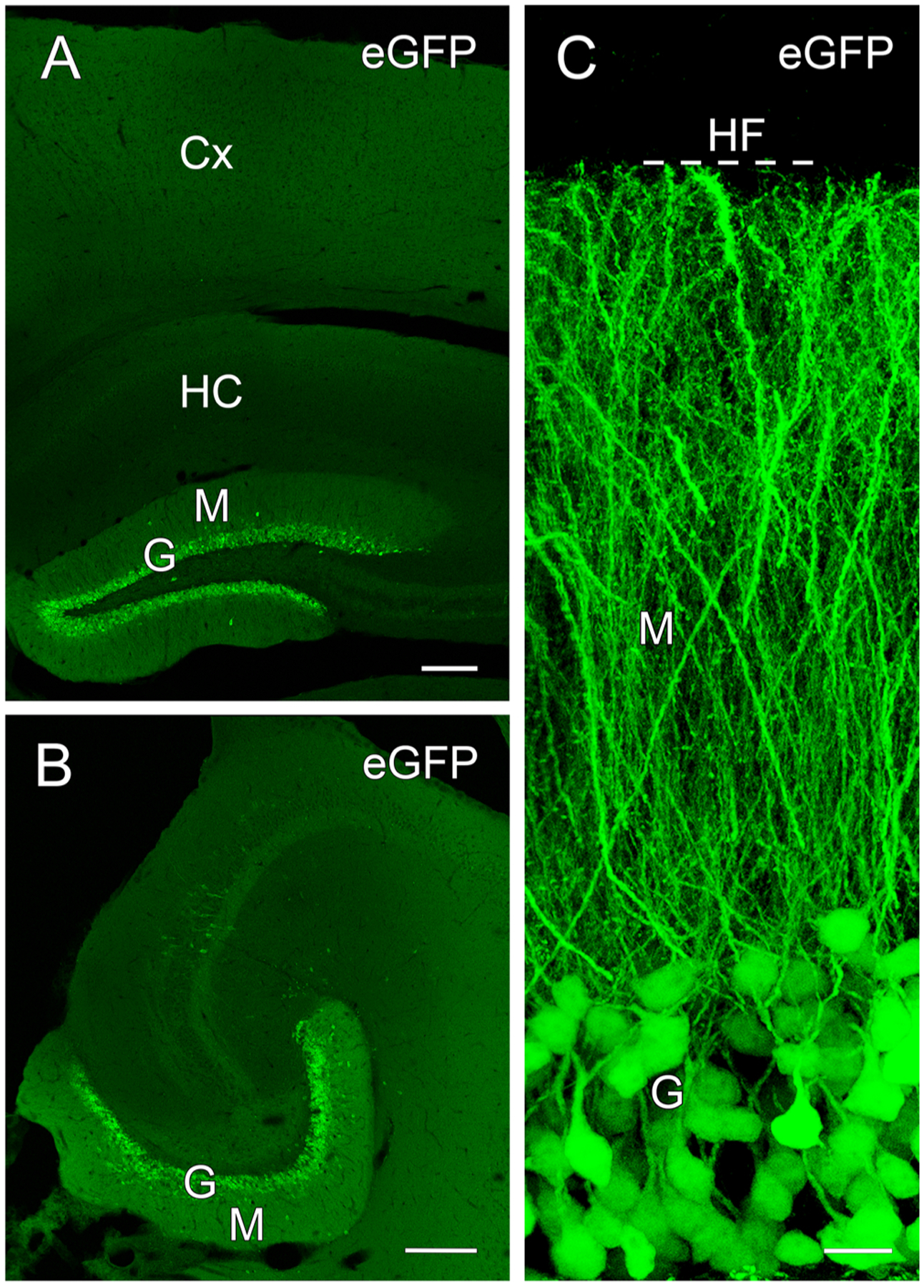
Dentate granule cells showed selective eGFP fluorescence at 1 month following transfection of eGFP-tagged δ subunit in the dentate gyrus of DOCK10-Cre mice. ***A,B***, The dentate granule cell (G) and molecular (M) layers were selectively labeled at both dorsal (*A*) and ventral (*B*) levels of the dentate gyrus. No specific labeling was evident in the hippocampus (HC) or cerebral cortex (Cx). ***C***, Our δ-eGFP construct was strongly expressed in the cell bodies of granule cells (G) and extended throughout their dendrites in the molecular layer (M), ending sharply at the hippocampal fissure (HF, dashed line). Scale bars: 200 μm (***A, B***) and 25 μm (***C***).

This study was designed to alter δ subunit expression selectively in dentate GCs, and thus it was important to verify that Cre expression in the DOCK10 mice was limited to dentate GCs and that these neurons could be labeled effectively with the current Cre-dependent constructs. Following viral transfection of either Cre-dependent δ subunit-eGFP or eYFP, dentate GCs were labeled with the fluorescent markers in both the rostral and caudal dentate gyrus (Fig. 2A,B). Strong labeling was evident in the cell bodies and extended throughout the granule cell dendrites in the molecular layer, ending sharply at the hippocampal fissure (Fig. 2C). Verifying strong dendritic labeling was important as dendrites in the molecular layer are the major location of GABA_A_R δ subunits in the dentate gyrus. No other regions were labeled in the hippocampal formation or cerebral cortex (Fig. 2A,B), demonstrating the high selectivity of the labeling for dentate GCs. Although some interneurons in the dentate and hippocampus were occasionally labeled, their numbers were very low compared to those of labeled GCs.

In our previous studies in the pilocarpine model of epilepsy in C57BL/6 mice, we identified changes in δ, α4 and γ2 subunit labeling in the dentate gyrus throughout the chronic period (Peng et al., 2004; Zhang et al., 2007). Similar changes were confirmed in nontransfected pilocarpine-treated DOCK10-Cre mice and were subsequently observed on the nontransfected side of the pilocarpine-treated animals (see later Results). Briefly, at 6–8 weeks following pilocarpine treatment in these mice, immunolabeling for the δ subunit was decreased throughout the molecular layer of the dentate gyrus. In a semiquantitative densitometry study of sections from control and pilocarpine-treated mice that had been processed together for immunohistochemistry, the mean decrease in δ subunit labeling in the pilocarpine-treated mice was 30.8% (n = 2 normal controls and 4 pilocarpine-treated; 4–6 sections per mouse). Sections that were processed in the same immunohistochemical run were also analyzed separately for all possible pairwise comparisons, and numerical values for the densitometric grey scale labeling ratios were significantly (p < 0.05) larger than unity at the lower end of the 95% confidence intervals in each of the 4 analyses (1.41, 1.58, 1.51, 1.43). In the same animals, α4 subunit labeling in the molecular layer was generally stronger in the pilocarpine-treated animals than in controls, and γ2 subunit labeling was also increased in the dentate molecular layer, as well as in the hippocampus, confirming our earlier findings in pilocarpine-treated mice (Peng et al., 2004).

**Fig. 3.**
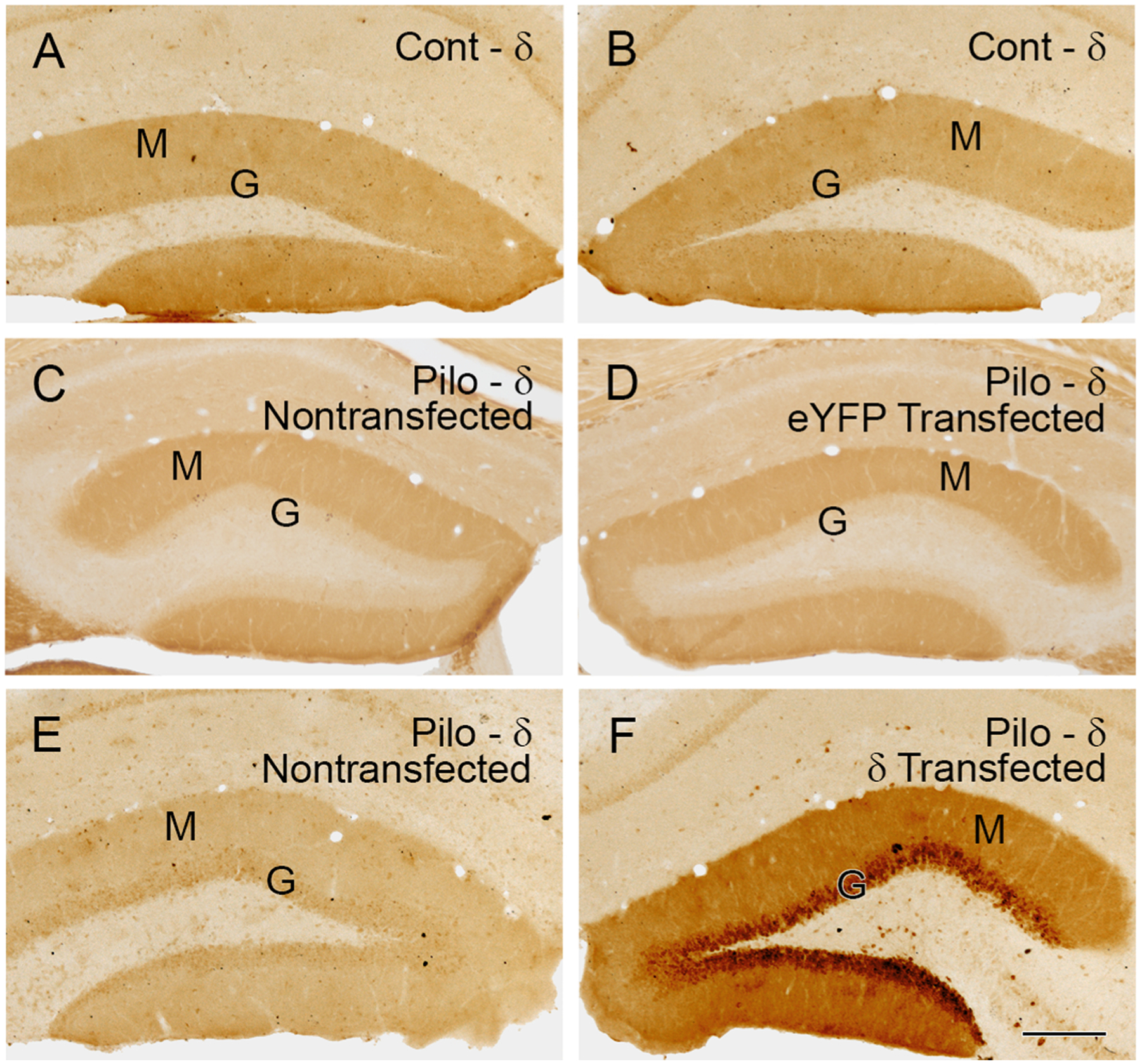
δ subunit expression was increased on the transfected side following unilateral transfection for the δ subunit in a pilocarpine (pilo)-treated DOCK10-Cre mouse. ***A,B***, In a nontransfected control (Cont) mouse, δ subunit labeling extends throughout the molecular (M) and granule cell (G) layers and the intensity of labeling is similar on the two sides. ***C,D***, In a pilo-treated mouse with unilateral eYFP transfection, the intensity of δ subunit labeling is also similar on the two sides and thus not altered by the eYFP transfection. ***E,F***, In a pilo-treated mouse with unilateral δ-eGFP transfection, δ subunit labeling is substantially higher in the granule cell layer (G) and throughout the molecular layer (M) on the transfected side (*F*) than on the nontransfected side (*E*). δ subunit labeling on the nontransfected side is lower than that in a control mouse (compare *A* and *E*). Scale bar: 200 μm (***A-D***).

Unilateral viral transfections of δ-eGFP or eYFP allowed comparisons of subunit labeling between the two sides in the same animal, and we confirmed that immunolabeling was comparable between the two sides of both nontransfected controls (Fig. 3A,B) and nontransfected pilocarpine-treated animals. Likewise, no side-to-side differences were evident in pilocarpine-treated animals with unilateral transfection of eYFP (Fig. 3C,D), indicating a lack of effect of eYFP transfection on δ subunit labeling in the granule cells. These findings provided a solid base for side-to-side comparisons of GABA_A_R subunit labeling in unilaterally-transfected animals.

### 3.2. δ subunit labeling was increased in dentate granule cells by viral transfection in DOCK10-Cre mice

Following viral transfection of the Cre-inducible δ subunit-eGFP construct in pilocarpine-treated DOCK10-Cre mice, δ subunit labeling was strongly increased on the transfected side (Fig. 3F). This δ subunit labeling was highest in the cell body layer but also extended throughout the molecular layer (Fig. 3F), as described previously for eGFP labeling. δ subunit labeling resulting from the transfection was substantially stronger on the transfected side than on the nontransfected side (compare Figs. 3E and 3F) and also often appeared stronger than that in control animals (compare Fig. 3F and A,B). Such labeling suggested a general overexpression of the δ subunit within the cytoplasm following Cre-dependent δ transfection. In the group of mice that was subsequently used for analyses of both α4 and γ2 labeling, semiquantitative analyses of optical density demonstrated a mean increase of 52.6% on the δ subunit- transfected side, ranging from 38.7% to 61.3% (n = 6 mice; 4–5 sections per mouse).

### 3.3. α4 subunit labeling was decreased on the δ subunit-transfected side of pilocarpine-treated mice

**Fig. 4.**
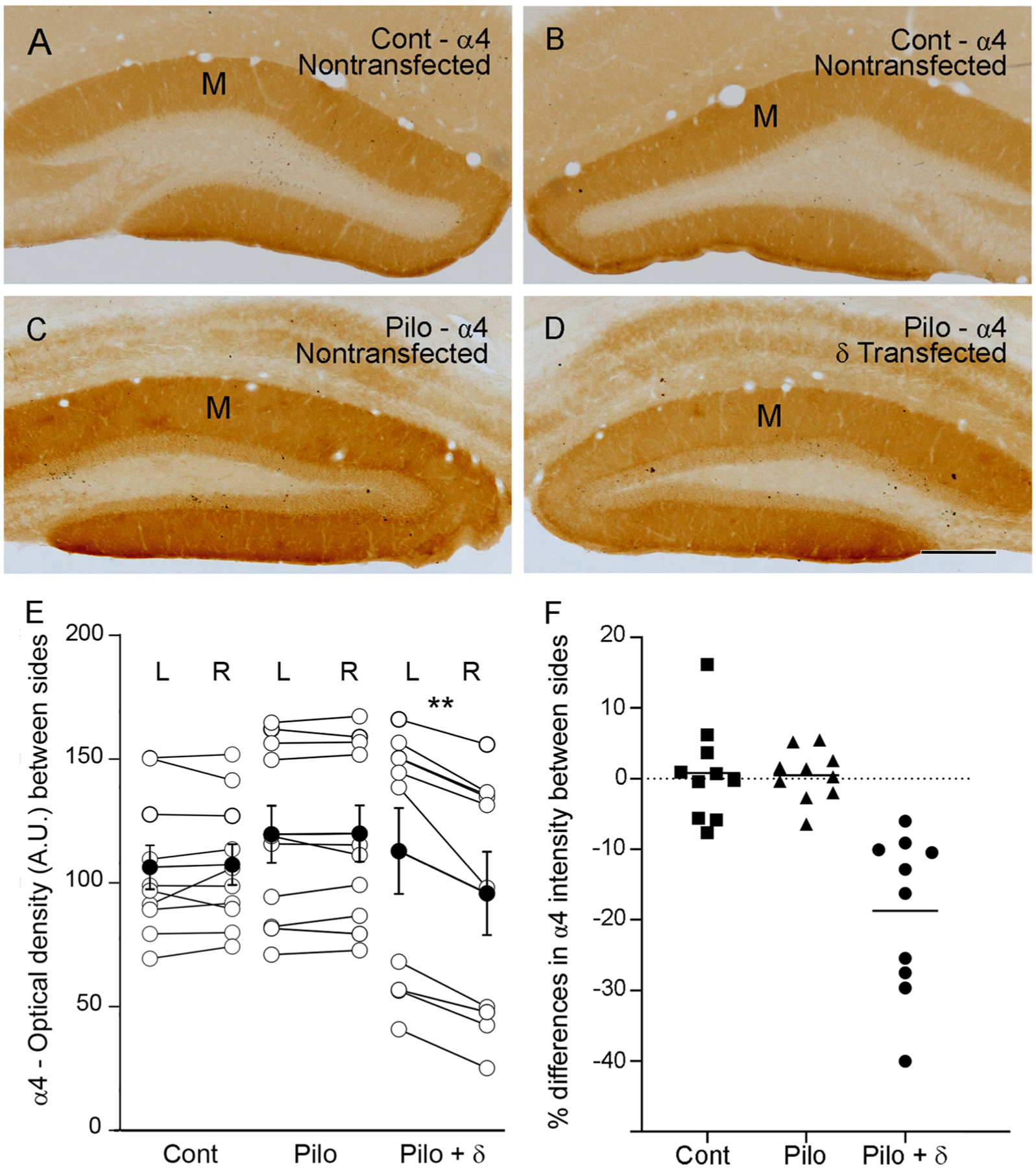
α4 subunit expression is decreased (downregulated) on the δ subunit-transfected side of a pilocarpine (pilo)-treated DOCK10-Cre mouse. ***A,B***, Comparison of the two sides of a control mouse showed similar levels of *α*4 labeling in the dentate molecular layer (M). ***C, D***, In a pilocarpine-treated mouse with unilateral δ transfection, α4 labeling in the molecular layer is higher on the nontransfected side (*C*) compared to that of a control mouse, but lower on the transfected side (*D*) than on the nontransfected side (*C*). The lower α4 expression on the transfected side suggests downregulation of the increased α4 expression that is observed in nontransfected pilo-treated mice. ***E***, Densitometry of α4 labeling in the dentate molecular layer showed a significantly greater difference in the intensity of labeling between the two sides in the δ subunit-transfected pilo-treated mice (**p = 0.001) than between sides of nontransfected control or pilo-treated mice. Only side-to-side comparisons within each group are relevant. Darker lines indicate the mean for each group. (n = 10 sections per group; 5 mice per group, 2 sections per mouse). ***F***, Percentage differences in the intensity of α4 labeling between sides are illustrated for each group. Consistent side-to-side differences were found only in pilo animals with unilateral δ subunit transfection. Solid lines indicate the means. Scale bar: 200 μm (***A-D***).

Immunolabeling for the α4 subunit was higher in the dentate molecular layer on the nontransfected side in pilocarpine-treated animals (Fig. 4C) compared to that in control animals (Fig. 4A,B), as described previously in pilocarpine-treated animals (Peng et al., 2004). However, following unilateral transfection of the δ subunit, α4 subunit labeling was consistently lower on the transfected side (Fig. 4D) than on the nontransfected side (Fig. 4C).

The intensity of α4 subunit labeling in the molecular layer was determined on each side with densitometry. Side-to-side comparisons were made in control animals, pilocarpine-treated animals with no transfection, and pilocarpine-treated animals with unilateral δ subunit transfection. All densitometry data were analyzed with a Wilcoxon paired two-tailed test. In both control animals and nontransfected pilocarpine-treated animals, the labeling on the two sides was not significantly different (p = 0.49 and 0.76, respectively; n = 10 per group; 5 mice, 2 sections per animal) (Fig. 4E), with a mean increase of 0.79% on the right side of control animals and 0.49% on the right side in nontransfected pilocarpine-treated animals (Fig. 4F). In contrast, α4 subunit labeling was significantly lower on the transfected side than on the nontransfected side of pilocarpine-treated animals (p = 0.001; n = 10; 5 mice, 2 sections per animal) (Fig. 4E), with a mean decrease of 18.75% on the transfected side (Fig. 4F). Because α4 labeling is typically increased in the molecular layer of pilocarpine-treated animals, the comparatively lower levels of labeling on the δ subunit-transfected side represented a downregulation of α4 subunit labeling on the transfected side compared to the nontransfected side.

### 3.4. γ2 subunit labeling was decreased on the δ subunit-transfected side of pilocarpine-treated mice

**Fig. 5.**
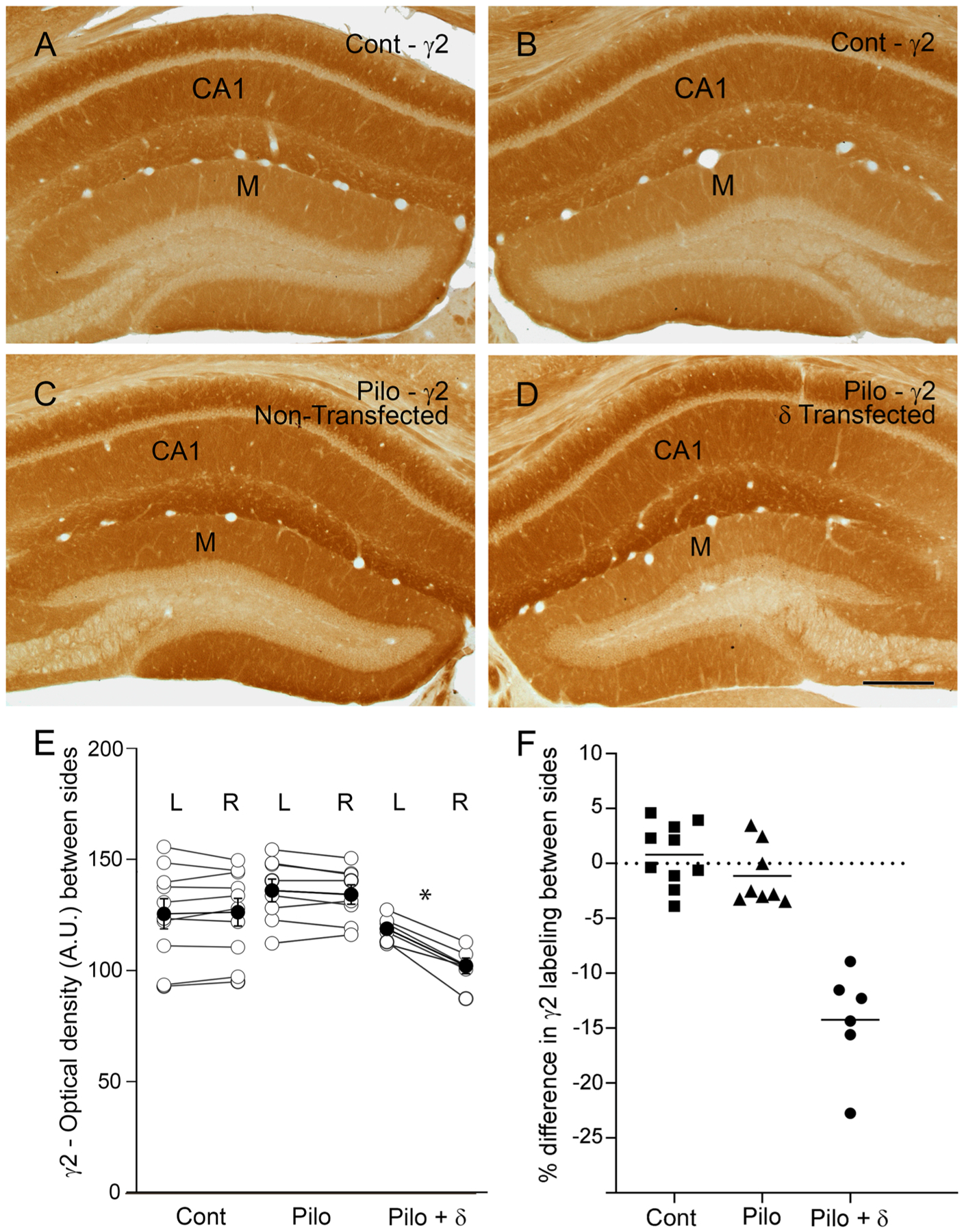
γ2 subunit labeling is decreased (downregulated) on the δ subunit-transfected side of a pilocarpine (pilo)-treated DOCK10-Cre mouse. ***A,B***, Comparison of the two sides of a control mouse showed similar levels of γ2 labeling in the dentate molecular layer (M). ***C***, In a pilocarpine-treated mouse, γ2 subunit labeling is higher in the molecular layer on the nontransfected side compared to that of the control mouse (compare *A* and *C*). ***D***, γ2 labeling in the molecular layer on the transfected side is lower than that on the nontransfected side (compare *C* and *D*) and is similar to the level of labeling in the dentate gyrus in a control mouse (compare *A*, *B* and *D*). The decreased (downregulated) γ2 subunit labeling appears restricted to the transfected molecular layer and is not evident in CA1 (compare *C* and *D*). ***E***, Densitometry of γ2 labeling in the molecular layer showed a consistently greater difference between the intensity of labeling on the two sides of the δ subunit-transfected mice than between sides of control and nontransfected pilo-treated mice (*p = 0.03). (n = 10 (control), 8 (pilo, nontransfected), 6 (pilo, transfected) sections; 5, 4, 3 mice per group respectively, 2 sections per mouse). Darker lines indicate the mean for each group. ***F***, Percentage differences in the intensity of γ2 labeling between sides are illustrated for each group. Consistent side-to-side differences were found only in pilo animals with unilateral δ subunit transfection. Solid lines indicate the means. Scale bar: 200 μm (***A-D***).

In control animals, the γ2 subunit is prominently localized in dendritic layers throughout the dentate gyrus and hippocampus, and levels of labeling were comparable on the two sides in control animals (Fig. 5A and 5B). In pilocarpine-treated animals, γ2 subunit expression was frequently higher in both the dentate gyrus and hippocampus (Fig. 5C) compared to that in control animals (Fig. 5A,B). Following δ subunit transfection in the dentate gyrus, γ2 subunit expression was decreased in the dentate molecular layer on the transfected side (Fig. 5D) compared to the nontransfected side (Fig. 5C). This decrease in γ2 labeling was restricted to the dentate molecular layer where δ subunit expression was increased and was not evident in the hippocampus that lacked Cre-dependent transfection of the δ subunit (compare Fig. 5C and 5D).

Densitometry demonstrated substantial side-to-side differences in the levels of labeling for the γ2 subunit in the δ subunit-transfected animals compared to those in both control and pilocarpine-treated mice without transfection (Fig. 5E,F). In control mice, the labeling on the two sides was not significantly different (p = 0.62; n = 10; 5 mice, 2 sections per mouse), with a 0.79% increase on the right side. Similarly, in pilocarpine-treated mice without transfection, there were no significant side-to-side differences, with a mean decrease of 1.14% on the right side. In the δ subunit-transfected mice, γ2 labeling was significantly decreased on the transfected side compared to the nontransfected side (*p* = 0.03; n = 6; 3 mice, 2 sections per mouse) (Fig. 5E), with a mean decrease of 14.24% on the transfected side (Fig. 5F). In view of the increased γ2 subunit expression in the pilocarpine-treated animals, the decrease in γ2 labeling in the molecular layer on the transfected side represented a downregulation of this subunit in the region of δ subunit transfection.

To determine if there was a correlation between the percentage increases in δ subunit labeling and the decreases in α4 and γ2 labeling, the densitometry data were analyzed with Spearman’s Rank-order Coefficient. We qualitatively observed that the greatest decreases in both α4 and γ2 occurred in sections with the greatest increases in δ subunit labeling. However, there was no statistically significant correlation (R = −0.66, p = 0.09 for δ and α4; R = −0.43, p = 0.19 for δ and γ2).

### 3.5. δ subunits were localized to the plasma membrane following δ subunit transfection

**Fig. 6.**
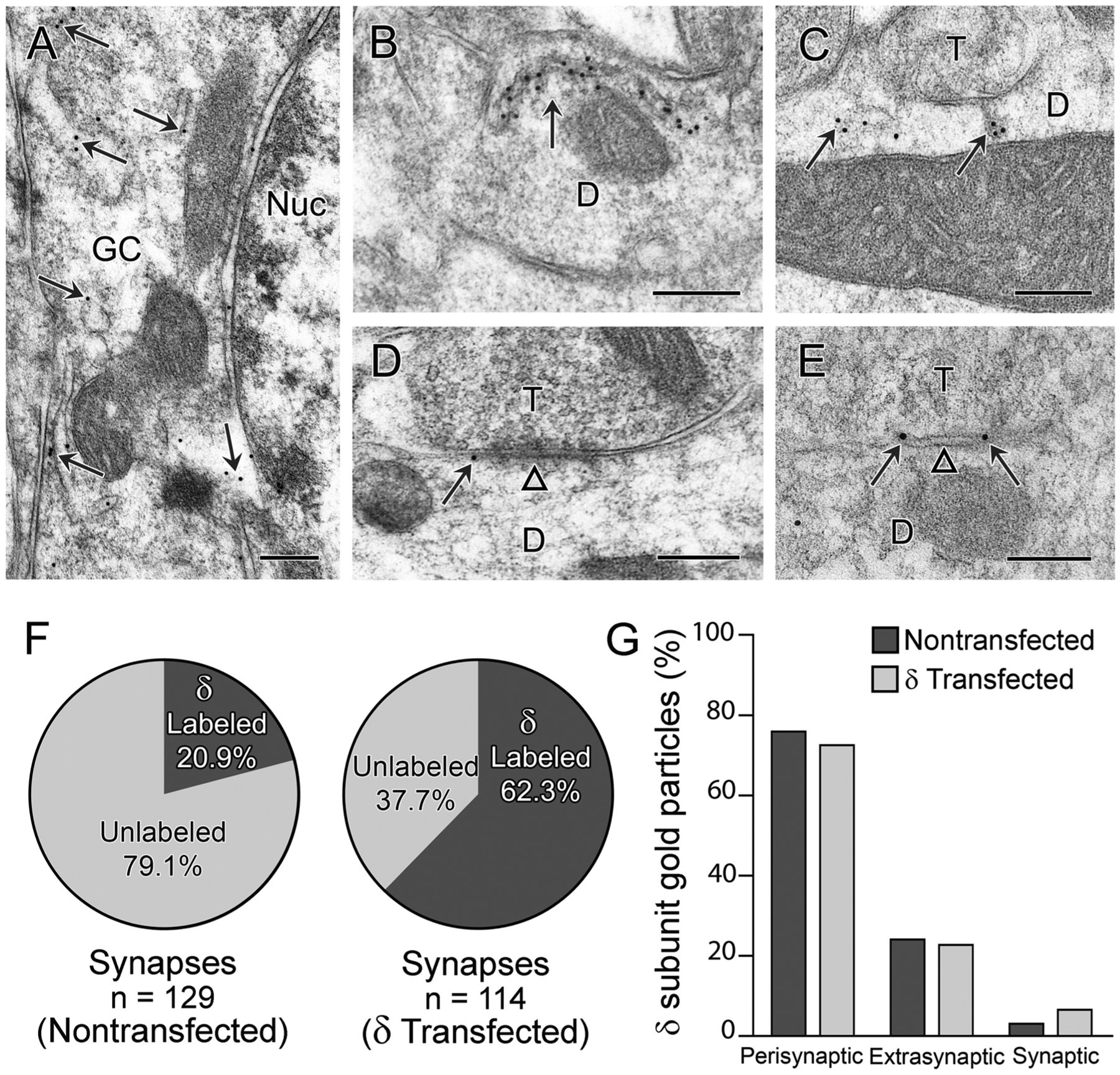
Immunogold labeling for the δ subunit is increased in granule cells on the transfected side of a pilocarpine (pilo)-treated mouse. ***A***, On the transfected side, immunogold particles (arrows) are abundant throughout the cytoplasm of the cell body of a granule cell (GC) but are not evident in the nucleus (Nuc). ***B***, Immunogold particles are concentrated within some endoplasmic reticulum in granule cell dendrites (D). ***C***, Gold particles are also evident within the dendritic cytoplasm near an axon terminal (T). ***D,E***, Immunogold particles are located at perisynaptic sites of symmetric synapses (open arrowheads) on granule cell dendrites. ***F***, The percentage of immunogold-labeled synapses was low on the nontransfected side but was substantially increased on the δ subunit-transfected side. ***G***, At labeled synapses, immunogold particles were located predominantly at perisynaptic locations on both the nontransfected and transfected sides. Scale bars: 0.20 μm (***A-E***).

Immunogold labeling for the δ subunit was increased in granule cells on the δ subunit-transfected side of the pilocarpine-treated mice, consistent with the increased labeling observed with light microscopy. Numerous immunogold particles were present in the cytoplasm of the GC somata and dendrites (Fig. 6A–C), and such cytoplasmic labeling was not observed on the nontransfected side. Immunogold labeling was also found at the plasma membrane on the transfected side, often in close proximity to symmetric synapses (Fig. 6D,E). At symmetric synapses with a clear postsynaptic contact, the percentage of δ subunit-labeled synapses was low on the nontransfected side (20.9%; n = 27 of 129 synapses) (Fig. 6F), consistent with previous findings in pilocarpine-treated C57BL/6 mice (Zhang et al., 2007). In contrast, the percentage of δ subunit-labeled synapses was substantially higher on the transfected side (62.3%; n = 71 of 114 synapses) (Fig. 6F). Despite this increase, the percentage of δ subunit-labeled synapses was lower than that found in a normal DOCK10 mouse (85.0%; 51 of 60 synapses) and in a previous study in control C57BL/6 mice (80.7%; Zhang et al., 2007). For the remaining labeled synapses on the nontransfected side, the percentage of gold particles was highest at perisynaptic sites (73.0%), with the remaining gold particles at extrasynaptic locations or directly at the synaptic contacts (24.3% and 2.7% respectively) (Fig. 6G). On the δ subunit-transfected side, the relative distribution of gold particles was very similar to that on the nontransfected side, with the highest percentage at perisynaptic locations (70.5%), and the remainder at extrasynaptic (23.0%) and synaptic (6.5%) locations (Fig. 6G). These findings demonstrate an increase in both cytoplasmic and plasma membrane localization of the δ subunit following δ subunit transfection and confirm that the preferential localization of the δ subunit at perisynaptic sites is maintained following transfection.

### 3.6. Neurosteroid insensitive tonic inhibition in pilocarpine-treated DOCK10 mice

**Fig. 7.**
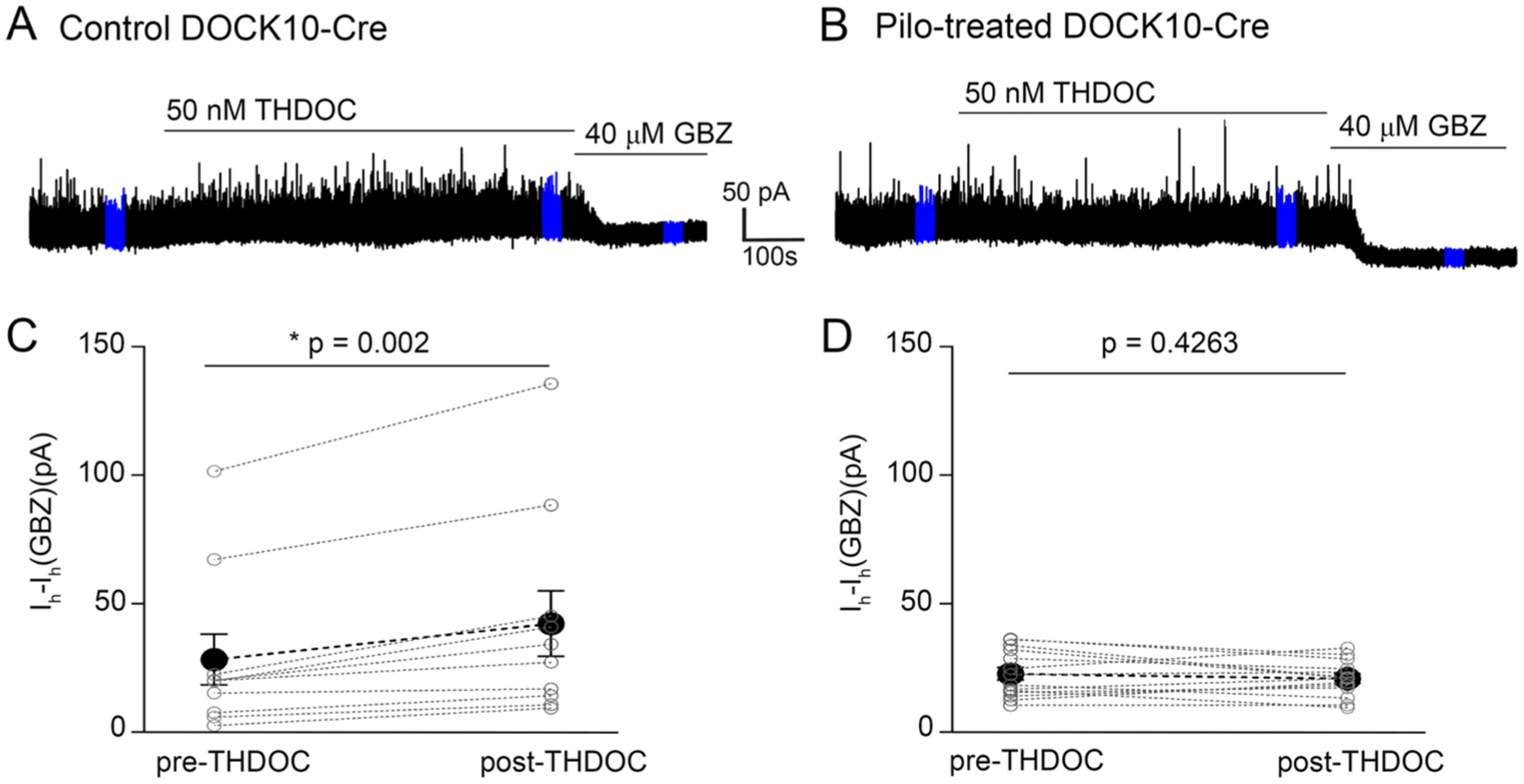
Loss of neurosteroid (THDOC) sensitivity of the tonic GABA conductance in pilocarpine (pilo)-treated DOCK10-Cre mice. ***A,B***, Illustration of raw recordings in two dentate GCs from control and pilo-treated mice. In each recording, the horizontal lines indicate the perfusion of 50 nM THDOC and 40 μM of the GABA_A_ receptor blocker gabazine (GBZ). The 30 s epochs used for the calculation of the tonic currents, as described in the Methods, are indicated in *blue*. ***C***, Plot of the paired values (pre-THDOC and post-THDOC) of the tonic currents in DOCK10-Cre mice (n = 2 mice, n = 10 slices). ***D***, Plot of the paired values (pre-THDOC and post-THDOC) of the tonic currents in pilo-treated DOCK10-Cre mice (n = 3 mice, n = 14 slices). The p-values indicated on the graphs were calculated according to a paired Wilcoxon test.

We have previously shown that following the decrease in GABA_A_R δ-subunits in pilocarpine-treated C57BL/6 mice, the tonic GABAergic inhibition of dentate gyrus GCs is compensated by the expression of most likely α4 and γ2 subunits (Zhang et al., 2007). However, as GABA_A_Rs without the δ-subunits show only modest modulation by neurosteroids, the characteristic neurosteroid-dependent enhancement of the dentate GC tonic GABA currents is missing in pilocarpine-treated mice. We wanted to ensure that this is also the case in the DOCK10 mice used in the present study. Fig. 7A,C shows the enhancement of tonic currents in dentate GCs of untreated DOCK10 mice, while Fig, 7B,D illustrates the loss of THDOC sensitivity in pilocarpine-treated DOCK10 mice. These electrophysiological findings corroborate our anatomical observations that GABA_A_R alterations in pilocarpine-treated DOCK10 mice replicate those previously reported in C57BL/6 mice (Zhang et al., 2007).

### 3.7. Enhanced tonic inhibition with regained neurosteroid sensitivity following δ subunit transfection in pilocarpine-treated DOCK10 mice

**Fig. 8.**
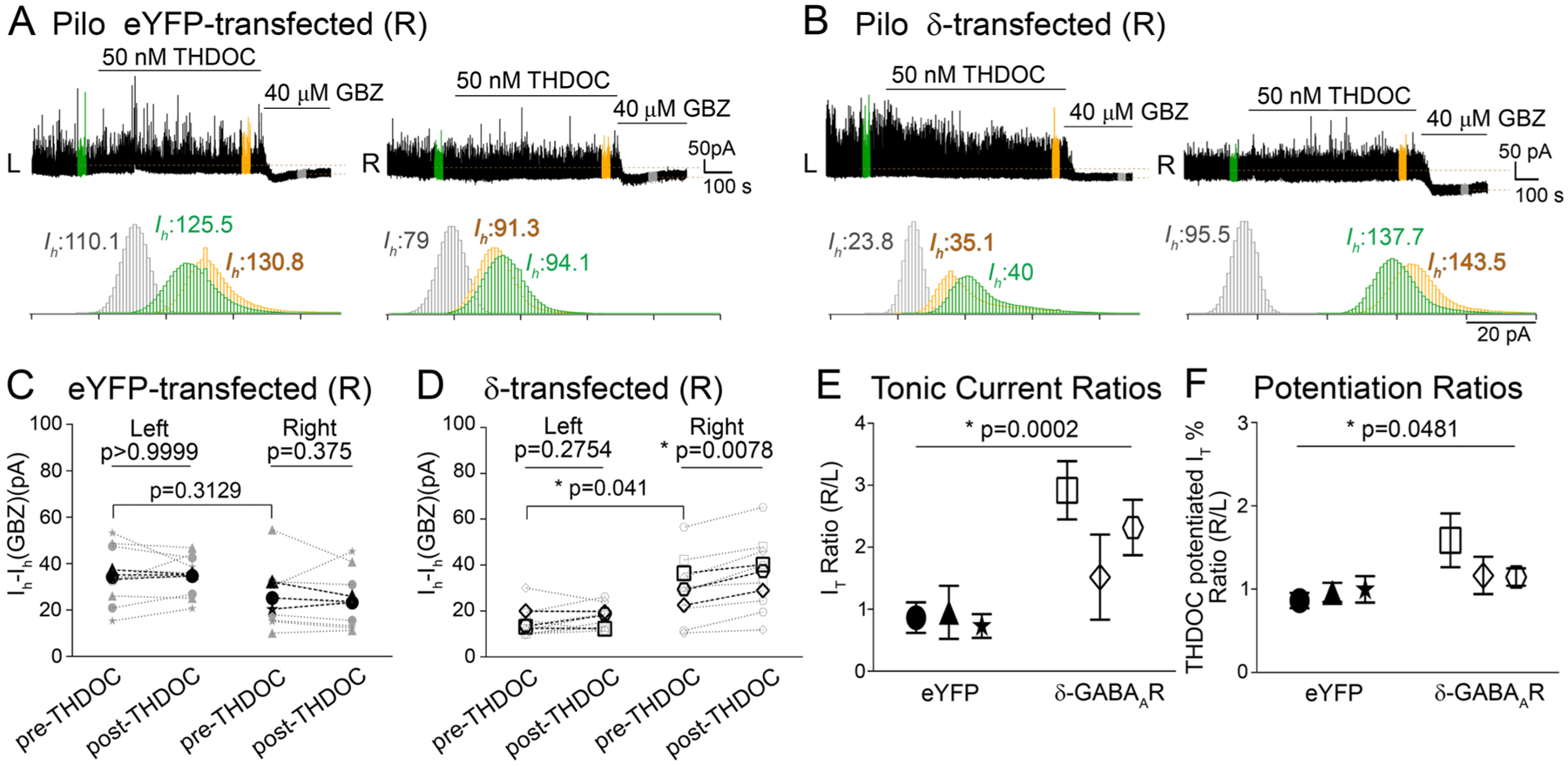
Unilateral (right side, R) viral transfection of GABA_A_R δ subunits increases the tonic GABA current and makes it regain its neurosteroid (THDOC) sensitivity in pilo-treated DOCK10-Cre mice. ***A***, Raw recordings of the tonic currents in two dentate GCs in the left (L, nontransfected) and right (R, transfected) dentate gyrus following viral transfection of eYFP. In each recording, the horizontal lines indicate the perfusion of 50 nM THDOC and 40 μM of the GABA_A_ receptor blocker gabazine (GBZ). The 30 s epochs used for the calculation of the tonic currents, as described in the Methods, are indicated as *green* during the pre-THDOC period, *orange* during the post-THDOC period, and *grey* during the GBZ perfusion period. Below each raw trace, all-points histograms show the distributions of holding current (I_*h*_).values during the color coded 30 s epochs. The mean I_*h*_ values are indicated above the distributions. ***B***, Raw recordings of the tonic currents in two GCs in the left (nontransfected) and right (transfected) dentate gyrus following viral transfection of GABA_A_R δ-eGFP. Color codes and I_*h*_ distributions as in ***A***. ***C,D***, Comparison between tonic currents recorded in the nontransfected (left) and transfected (right) sides in dentate GCs after eYFP (***C***, n = 7 cells on each side, n = 3 mice) or δ-eGFP (***D***, n = 7 cells on left and n = 8 cells on right, n = 3 mice) transfections. Connected symbols indicate paired comparisons between pre- and post-THDOC tonic current values. The pre-THDOC tonic current values were also compared between the nontransfected (left) and transfected (right) using unpaired comparisons. The p-values are indicated above the lines connecting the compared groups as obtained following the Wilcoxon paired or unpaired tests. The three symbol shapes indicate the averages of the tonic current values for the three mice in each group (eYFP- or δ-transfected). ***E***, Average ratios of pre-THDOC tonic currents calculated in each mouse by averaging the ratios obtained by dividing every tonic current amplitude recorded in dentate GCs on the transfected (R) side by each of the values recorded on the nontransfected (L) side. Symbol codes for the three animals in each group as in ***C*** and ***D***. The p-value was obtained by the Mann-Whitney unpaired test. ***F***, Comparison of the relative potentiation of the tonic currents by 50 nM THDOC expressed as a ratio of the potentiation measured in the transfected (R) side divided by that observed in the nontransfected (L) side. The values represent the averages for each of the three mice in each group and are symbol-coded as in ***E***. The p-value was obtained by the Mann-Whitney unpaired test.

Our anatomical findings are consistent with the renewed expression of GABA_A_R δ subunits following unilateral transfection in the dentate gyrus of pilocarpine-treated DOCK10 mice. In order to ascertain whether these receptors constitute functional GABA-gated ion channels responsible for tonic inhibition, we performed whole-cell patch clamp recordings from dentate gyrus GCs in pilocarpine-treated animals following unilateral transfection of either eYFP or δ subunit-eGFP. On the transfected side, recordings were made only from fluorescent cells to ensure that the transfections were successful in the recorded neurons. In a raw trace of the recorded currents, Fig. 8A shows that there were no differences in the tonic currents recorded between the side transfected with eYFP virus (R) and the nontransfected (L) side. Moreover, the THDOC sensitivity of the tonic inhibition was absent, just as in the pilocarpine-treated DOCK10-Cre mice that received no viral transfection (see Section 3.6). Fig. 8B shows similar recordings in GCs from slices on the nontransfected (L) and GABA_A_R δ-subunit transfected (R) sides of a pilocarpine-treated mouse. As indicated by the all-points histograms, the tonic current is larger on the transfected side, and it is further potentiated by 50 nM THDOC. Fig. 8C and D show the summary of our findings in three animals per group, each transfected on the right side with eYFP or GABA_A_R δ-subunits. The averages of the tonic current ratios obtained by dividing the current amplitudes from the transfected sides with those from the nontransfected sides in a given animal are illustrated in Fig. 8E. All three animals transfected with δ subunits had a larger tonic GABA current on the side of the transfection, which was significantly different than the same ratio in eYFP-transfected animals. The expression of GABA_A_R δ-subunits on the transfected side resulted in a nearly 2–3 fold larger current on the transfected side when compared to the eYFP-expressing animals (p = 0.0002, Mann-Whitney test). As evidence that the increased tonic currents were mediated by GABA_A_R-containing δ subunits, the sensitivity of the tonic currents to the neurosteroid THDOC was once more detectable as indicated by the larger R/L ratios of the THDOC-induced potentiation of the tonic currents in the δ subunit-transfected animals compared to the eYFP-transfected mice (Fig. 8F). There were no differences noted in the magnitude of the phasic currents between any of the groups. Moreover, THDOC at 50 nM did not have significant effects on phasic currents, as the sensitivity of the synaptic events is much lower than that of the tonic GABA conductance (Stell et al., 2003). Our findings on the tonic currents clearly indicate the effectiveness of the GABA_A_R δ-subunit transfections and indicate the formation of functional GABA_A_Rs by the transfected subunits.

### 3.8. Reduced dentate gyrus network excitability following δ subunit transfection in pilocarpine-treated DOCK10 mice

We next wanted to know whether the increased expression of δ subunit-containing GABA_A_Rs and the enhanced tonic currents in pilocarpine-treated mice on the side of the transfection were also manifest in reduced excitability of the dentate gyrus as measured by the stimulus-response curves in field EPSP (fEPSP) recordings. We have previously used such stimulus-response curves of perforant path-evoked fEPSPs recorded in the dentate gyrus to demonstrate changes in the excitability of the dentate network in pilocarpine-treated mice and the altered effects of THDOC in this rodent model of temporal lobe epilepsy (TLE) (Peng et al., 2004).

**Fig. 9.**
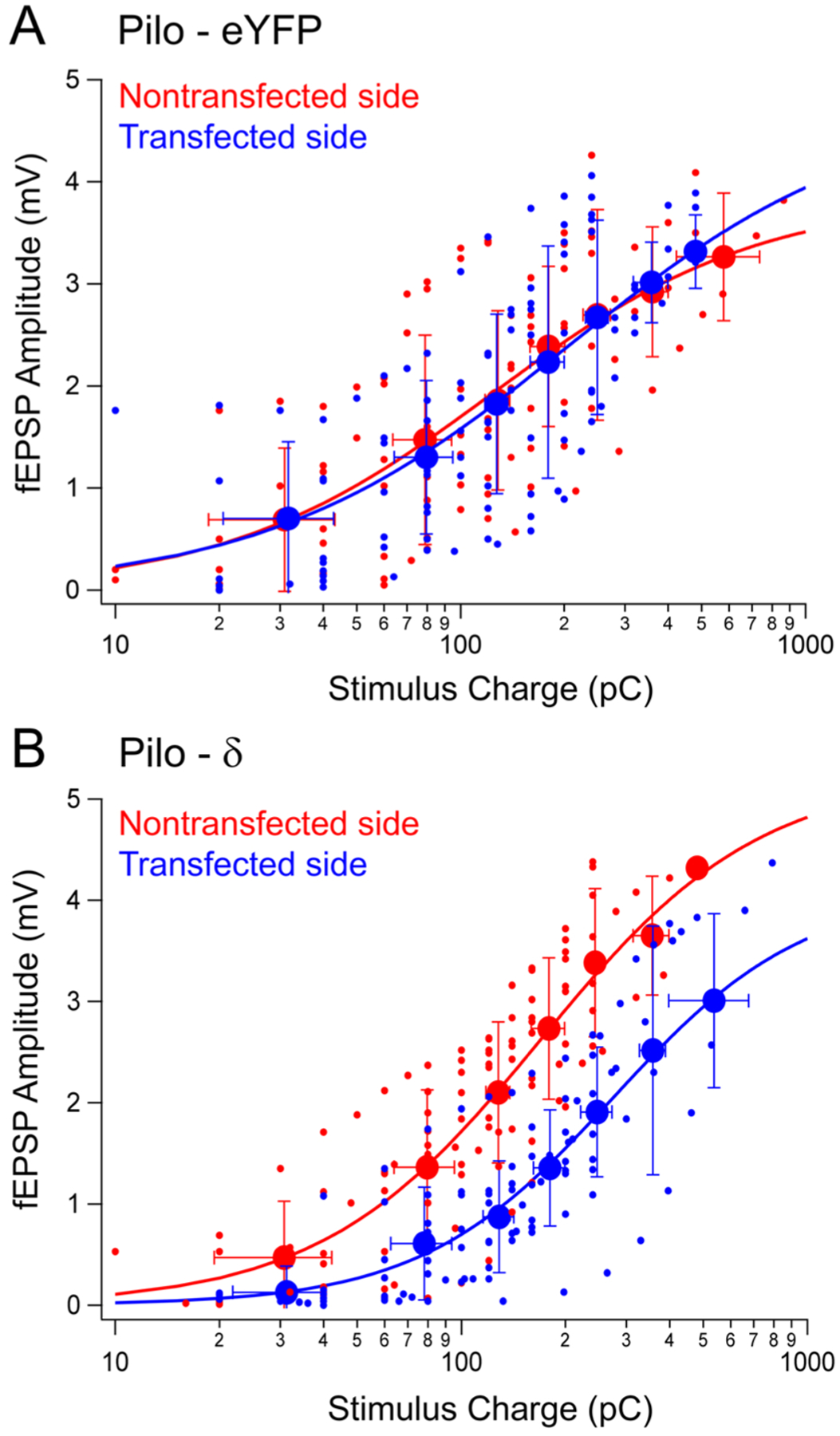
Stimulus-response curves in pilocarpine-treated DOCK10-Cre mice expressing no viral transcript (*red*) on the left (L) side, and either eYFP or δ-eGFP in the GCs of the right (R) dentate gyrus (*blue*). The absolute fEPSP amplitudes were plotted as a function of stimulus charge (Q in pC, calculated as the product between the stimulus current intensity and stimulus duration). The plots show the average values binned by consecutive Q values (5–10 responses/bin, large symbols with error bars as SD in both x and y direction). The continuous curves are fitted Hill equations to the averaged values. The small symbols indicate all the values obtained from the individual experiments. ***A***, The viral expression of eYFP on the R side (*blue*) did little to change the stimulus-response curves in pilocarpine-treated DOCK10-Cre mice (n = 8 slices, n = 3 mice for L; n = 9 slices, n = 3 mice for R). The two fitted Hill curves are not significantly different, (t-test p = 0.98 for the Q50 values and p = 0.403 for the Hill coefficients values between the two fitted curves). ***B***, Absolute values of fEPSP amplitudes and their SD plotted as in **A**, showing a significant reduction in perforant path-induced excitability of granule cells expressing δ subunits in pilocarpine-treated DOCK10-Cre mice (n = 11 slices, 4 mice for L; n = 9 slices, 3 mice for R), as reflected by the significant shift to the right in the Q50 values from 155.6 ± 6.8 pC in the nontransfected side to 340.4 ± 20.1 pC on the transfected side (t-test p = 3.83 ×10^−14^) while there was no change in the Hill coefficients (t-test p = 0.825).

The unilateral expression of eYFP alone in pilocarpine-treated mice did not significantly alter the excitability (the mean ± SEM values obtained from the fitted Hill equations were Q50 = 220.5 ± 23.3 pC, Hill coefficient = 0.86 ± 0.11 for the eYFP transfected side and Q50 = 219.9 ± 19.9 pC, Hill coefficient = 1.00 ± 0.13 for the nontransfected side, yielding a t-value of 0.021 with p = 0.98 for the Q50 comparison and a t-value of 0.839 with p = 0.403 for the Hill coefficient comparison (Fig. 9 A); indicating that viral transfection with a fluorescent protein alone did not change the altered neuronal excitability in the epileptic dentate gyrus. In sharp contrast, the expression of δ subunits on the transfected side considerably reduced the excitability of the network in the dentate gyrus (the mean ± SEM values obtained from the fitted Hill equations were Q50 = 340.4 ± 20.1 pC, Hill coefficient = 1.49 ± 0.13 for the δ subunit transfected side and Q50 = 155.6 ± 6.8 pC, Hill coefficient = 1.45 ± 0.12 for the nontransfected side, yielding a t-value of 8.714 with p = 3.83 ×10^−14^ for the Q50 comparison and a t-value of 0.222 with p = 0.825 for the Hill coefficient comparison (Fig. 9B).

When compared to the nontransfected side, where the expression of δ subunits was reduced, the viral transfection-induced increase in the expression of these subunits resulted in a significant decrease in network excitability. The stimulus-response curves showed significantly less excitability in pilocarpine-treated animals on the transfected side when compared to the contralateral, nontransfected side (Fig. 9B). The average value of the fEPSP amplitudes evoked by the largest stimuli was reduced by 30%, from 4.32 mV on the nontransfected side to 3.01 mV on the δ subunit-transfected side. These data are consistent with the induced expression of δ subunit-containing GABA_A_Rs resulting in a lower level of neuronal excitability in the dentate gyrus.

### 3.9. Reduced dentate gyrus network excitability following δ subunit transfection is due to the expression of THDOC-sensitive GABA_A_Rs

**Fig. 10.**
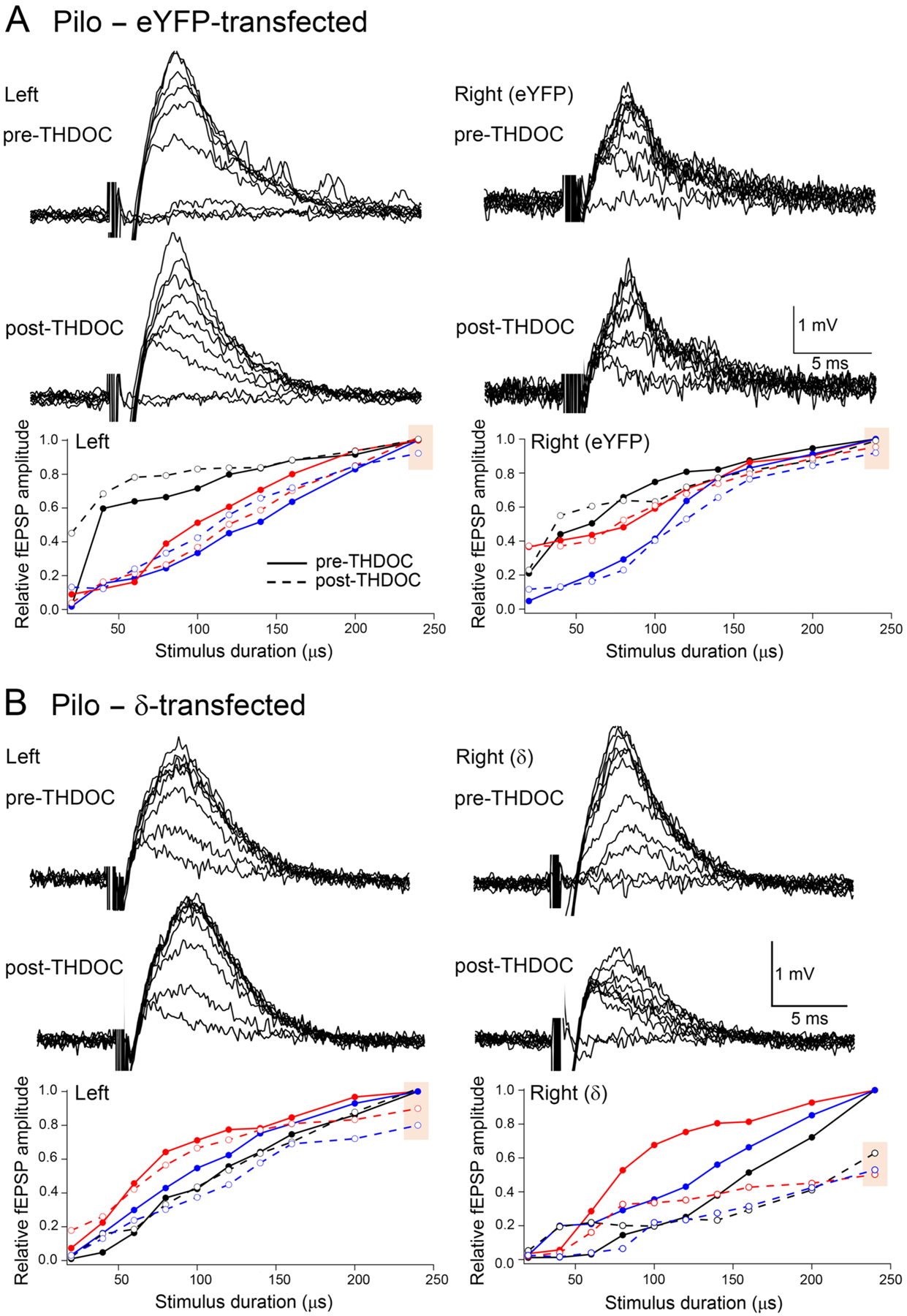
Raw fEPSP traces and related stimulus-response curves evoked by perforant path stimulation in pilocarpine-treated DOCK10-Cre mice expressing either eYFP (Panel ***A***) or δ-eGFP (Panel ***B***) in GCs of the right (R) dentate gyrus. The raw fEPSP traces recorded in the GC layer (upper sections of ***A*** and ***B***) were evoked by the various stimulus durations (20–240 μs) in two separate slices (L and R) perfused together in the recording chamber and exposed simultaneously to 20 nM THDOC. (The illustrated raw traces were obtained from animals represented by the red stimulus-response curves below). Stimulus artifacts have been partially removed for clarity. The pre-THDOC labeled traces were obtained just before the start of the THDOC perfusion while the post-THDOC traces were collected starting at 10 min into the THDOC perfusion. The lower panels of ***A*** and ***B*** show stimulus-response curves from all 3 pilocarpine-treated mice in each group (color-coded black, blue or red). The responses were averaged from 3 to 4 slices (from L and R) that were perfused together in the recording chamber as described above. The pre-THDOC labeled traces (solid symbols connected by solid lines) were obtained just before the start of the THDOC perfusion while the post-THDOC traces (open symbols connected by dashed lines) were collected starting at 10 min into the THDOC perfusion. Before averaging, the fEPSP amplitudes were normalized in each slice to the largest pre-THDOC amplitude evoked by a stimulus duration of 240 μs. In each slice, the post-THDOC values were also normalized to the corresponding largest pre-THDOC amplitude value before averaging. The post-THDOC values evoked by the largest stimulus (240 μs; pink boxes) were compared using paired statistics between the corresponding left and right slices (see text for details). THDOC produced a significant reduction in these fEPSP amplitudes only on the right side transfected with δ subunits, indicating that functional neurosteroid-sensitive δ subunit-containing GABA_A_ receptors were restored in the pilo-treated dentate GCs. The averages for each group of these mice are shown in Fig. 11.

We next examined whether it was the neurosteroid-sensitive δ subunit-containing GABA_A_Rs that were responsible for the decreased dentate network excitability on the transfected side. Therefore, we perfused 20 nM THDOC after stable baseline fEPSP responses were recorded and a pre-THDOC stimulus-response curve was obtained. At the end of a 10 min perfusion with THDOC, the stimulus-response curve of the fEPSPs was repeated. In most experiments, we used two slices in the same recording chamber. One slice was from the transfected side, the other from the nontransfected side. Examples of the raw traces from such a recording in an eYFP-transfected pilocarpine-treated animal are illustrated in Fig. 10A (compare upper and lower traces for each side). On each side, the effect of THDOC is unremarkable, as illustrated by the normalized stimulus-response curves below the raw traces. These stimulus-response curves were obtained from the three animals in this group (color-coded as black, blue and red), and the raw traces were obtained from the animal represented by the red trace. Another pair of slices from an animal transfected with δ subunit-eGFP on the right side is shown in Fig. 10B. The raw traces (compare upper and lower traces) and stimulus-response curves from the three animals in this group show a clear inhibitory effect of 20 nM THDOC on the δ subunit-transfected side.

**Fig. 11.**
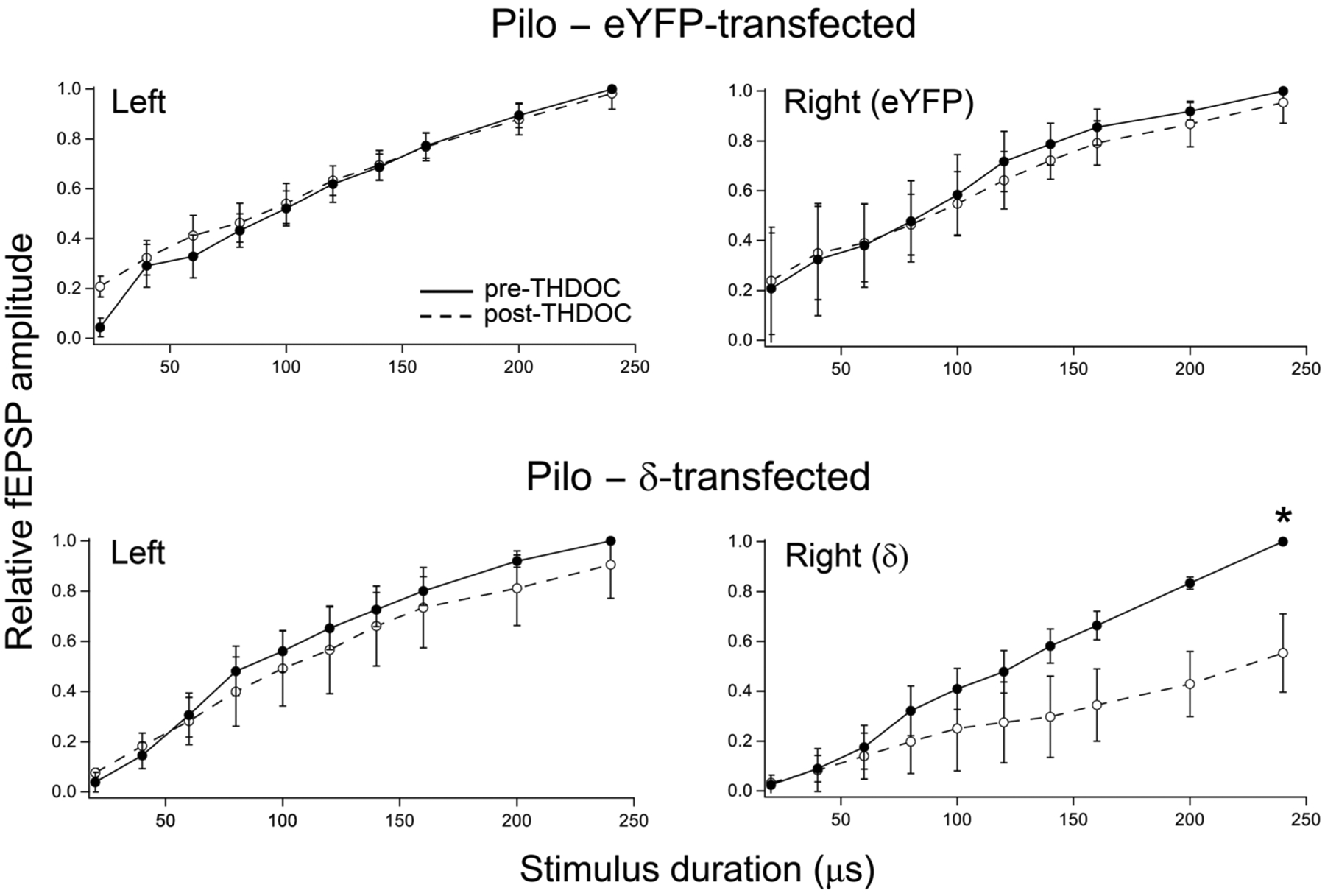
Averaged stimulus-response curves in pilocarpine-treated DOCK10-Cre mice expressing either eYFP (n = 3) or δ-eGFP (n = 3) in the GCs of the right dentate gyrus. (Averages of data from the individual mice shown in Fig. 10). In each mouse, the response used to calculate the averages was itself an average obtained from 3 to 4 slices, each from L and R, that were perfused together in the recording chamber and exposed simultaneously to 20 nM THDOC. The pre-THDOC labeled traces (solid symbols connected by solid lines) were obtained just before the start of the THDOC perfusion while the post-THDOC traces (open symbols connected by dashed lines) were collected starting at 10 min into the THDOC perfusion. Before averaging, the fEPSP amplitudes were normalized in each slice to the largest pre-THDOC amplitude evoked by the longest stimulus duration of 240 μs. In each slice, the post-THDOC values were also normalized to the corresponding largest pre-THDOC amplitude value before averaging. The post-THDOC values evoked by the largest stimulus (240 μs) were compared using paired statistics between the corresponding left and right slices (see text for details). THDOC produced a significant reduction (*p = 0.013) in the averaged fEPSP amplitudes only on the right side (δ-transfected), indicating that functional neurosteroid-sensitive δ subunit-containing GABA_A_ receptors were once again present in the pilo-treated dentate GCs.

The summary of these experiments is shown on Fig. 11. There are 3 animals in each group, with the stimulus-response curves representing the average values for all the slices (n = 3–4 per animal) in a given animal. The normalized fEPSP responses to the largest stimulus-evoked fEPSPs were compared before and after THDOC exposure. We used paired statistics for the nontransfected vs the transfected sides between the post-THDOC values for each animal. These paired values were 1.007 vs 0.990; 1.017 vs 0.953; and 0.923 vs 0.919 for the nontransfected vs the eYFP-transfected sides, respectively. The p-value of the paired *t*-test is 0.254, indicating no differential effect of THDOC between the two sides. In contrast, for the δ subunit-eGFP-transfected animals these values were: 1.017 vs 0.629; 0.899 vs 0.502; and 0.800 vs 0.529, yielding a paired *t*-test p-value of 0.013. This indicates that THDOC significantly decreased the dentate gyrus network excitability on the transfected side, thus demonstrating that it was the presence of functional neurosteroid-sensitive δ subunit-containing GABA_A_Rs that was responsible for the decrease in excitability.

## 4. Discussion

The key findings of this study are that 1) viral transfection of Cre-inducible GABA_A_R δ subunits can increase δ subunit expression in dentate GCs in an epilepsy model in which functional δ subunits are decreased and the disease process is ongoing; 2) increasing the expression of δ subunits also reinstated more normal levels of the α4 and γ2 GABA_A_R subunits in this model; and 3) the unilateral viral expression of functional δ subunit-containing receptors, but not of eYFP alone, leads to increased sensitivity to the neurosteroid THDOC and decreased dentate GC excitability.

Some increase in δ subunit labeling in Cre-expressing GCs was expected following the transfection, based on our previous studies of δ subunit transfection in normal somatostatin-Cre mice (Tong et al., 2015). However, it was unclear how robust the expression would be and whether functional GABA_A_Rs would be formed in the pathological conditions of an animal model of epilepsy in which a decrease in δ subunit levels had already occurred and ongoing epilepsy-related processes could counteract the formation of new δ subunit-containing receptors. Despite the adverse conditions, the δ subunit was strongly expressed, with an increase in labeling along the plasma membrane, including perisynaptic sites, on the δ-transfected side of the pilocarpine-treated animals.

In the δ-transfected animals, there were often higher than normal levels of labeling within the cytoplasm of both the cell bodies and dendrites of dentate GCs, and such cytoplasmic labeling suggested overexpression of the subunit. This strong labeling could be related to a general lack of control of expression levels for virally-delivered constructs. However, excess GABA_A_R subunits are normally degraded and removed from the cell (Luscher et al., 2011; Macdonald and Kang, 2012), and thus additional factors related to δ subunit regulation could contribute. For example, the apparent excess of δ subunits in the cytoplasm could reflect the relatively slow degradation of the δ subunit which has been noted following its transfection in HEK293 cells (Botzolakis et al., 2016). We also cannot rule out the possibility that fusion with eGFP could limit trafficking, assembly and surface expression of the δ subunit and thus contribute to retention of some subunits within the cytoplasm. However, the surface expression and normal localization that were observed make this possibility less likely.

The functional effects of high levels of the δ subunit in the GCs are not known, but excess accumulation of the GABA_A_R subunits could overwhelm the normal degradation processes and be detrimental to the stability of the cells (Botzolakis et al., 2016; Lagrange et al., 2018). The integrity of the labeled granule cells did not appear to be compromised over the time course of the current study, but the high levels of δ subunit in the cytoplasm call attention to the potential need to regulate subunit expression following such interventions. Although the ratios of GABA_A_R subunits cannot be controlled in vivo, as is possible in cell lines, recent studies of recombinant receptors have suggested that transfection of low levels of the δ subunit DNA, compared to the levels for the α and β subunits, may be sufficient for optimal assembly of α1/β/δ receptors in HEK293 cells (Botzolakis et al., 2016). Thus, lower levels of AAV transfection might have been equally or more effective in increasing δ subunit expression to appropriate levels in the current study. Alternatively, some overexpression might be necessary for the δ subunit to compete successfully for partnership with other subunits and form functional receptors.

While our light microscopic findings demonstrated robust expression of the δ subunit, ultrastructural studies were required to demonstrate increased δ subunit labeling at the plasma membrane on the transfected side compared to that on the nontransfected side. Immunogold labeling confirmed the surface localization of the δ subunit as well as its predominant localization at perisynaptic or extrasynaptic sites on the transfected side, consistent with the normal localization of this subunit (Nusser et al., 1998; Wei et al., 2003; Zhang et al., 2007). This non-synaptic location of δ subunit-containing receptors appears to be maintained in multiple conditions and may be related, in part, to restricted diffusion of these receptors, possibly resulting from interactions of the δ subunit with unknown intracellular partners (Hannan et al., 2020) and a lack of association with synapse targeting complexes (Martenson et al., 2017). A similar nonsynaptic localization has been found in previous GABA_A_R subunit transfection studies of the δ subunit in hilar somatostatin neurons (Tong et al., 2015) and the α6 subunit in CA1 pyramidal cells (Wisden et al., 2002).

The observed increase in surface expression on the transfected side (compared to the nontransfected side) of the pilocarpine-treated animal suggests that the δ subunit assembled with other GABA_A_R subunits, as is generally required for trafficking to the cell surface (Lorenz-Guertin and Jacob, 2018; Luscher et al., 2011, for reviews). The α4 subunit appears to be the most likely partner, considering the preferential partnership of the δ and α4 subunits in principal cells of the forebrain (Sur et al., 1999), the similar locations of α4 and δ in the dentate gyrus (Peng et al., 2002; Pirker et al., 2000), and the abundance of the α4 subunit, due, in part, to the increased α4 expression in dentate GCs in this epilepsy model (Brooks-Kayal et al., 1998; Peng et al., 2002). Although the γ2 subunit is also abundant in the dentate gyrus and increased in this model, previous studies have noted that the δ and γ2 subunits are mutually exclusive in native GABA_A_Rs (Araujo et al., 1998; Olsen and Sieghart, 2009).

Partnership of the δ and α4 subunits, along with a β subunit, may also have influenced the trafficking and cell surface expression of the δ subunit. In a mouse L(-tk) fibroblast cell line, the δ subunit did not reach the cell surface unless both α4 and β subunits were also expressed (Brown et al., 2002). Likewise, in a study of α4-deficient mice, the δ subunit remained sequestered in the cytoplasm of neuronal cell bodies in the thalamus and exhibited very little expression on dendrites in the neuropil, suggesting that the α4 subunit was necessary for surface expression of the δ subunit in this region (Peng et al., 2014). Partnership of the δ and α4 subunits could thus be critical for cell surface expression of the δ subunit in the current study, and further studies of the role of the α4 subunit in trafficking of the δ subunit are needed.

A major finding of the present study is that transfection of the δ subunit alone, to compensate for their reduced levels in epileptic animals, leads to more normal expression of the α4 and γ2 subunits in precisely the regions in which δ subunit labeling was increased. In this epilepsy model, as well as other models, α4 and γ2 subunit levels are increased in the dentate gyrus during the chronic period (Peng et al., 2004; Rajasekaran et al., 2010; Schwarzer et al., 1997). Following selective transfection of the δ subunit in dentate GCs, immunolabeling for the α4 and γ2 subunits was reduced specifically in granule cell dendrites in the molecular layer. In contrast, the elevated γ2 labeling in CA1 was not altered, demonstrating a clear relationship between the γ2 subunit changes and the δ subunit transfection. Such changes in response to transfection of a single GABA_A_R subunit have not been demonstrated previously and were not necessarily expected. In an earlier study in which α1 subunit levels were increased in the dentate gyrus through viral transfection in a rat pilocarpine model, changes in levels of other subunits were not described, and there were no significant differences in mRNA levels for any GABA_A_R subunits other than α1 (Raol et al., 2006).

Alterations in multiple subunits in the dentate gyrus in the current epilepsy model has suggested that the subunit composition of remaining GABA_A_Rs is altered. When the δ subunit is decreased in pathological conditions, partnership of the γ2 and α4 subunits appears to increase, and parallel increases in these subunits have been noted in several animal models of epilepsy, traumatic brain injury and alcohol withdrawal (Boychuk et al., 2016; Olsen and Spigelman, 2012; Peng et al., 2004; Rajasekaran et al., 2010). In addition, co-precipitation studies have demonstrated an increased association of the α4 and γ2 subunits in epilepsy models (Lund et al., 2008; Rajasekaran et al., 2010), supporting the suggestion that α4 and γ2 are assembled in the same GABA_A_R. Likewise, in the δ subunit-deficient mouse, γ2 subunit labeling increases substantially in the same regions in which the δ subunit is normally expressed, presumably partnering with the α4 subunit (Peng et al., 2004). Such changes likely reflect robust homeostatic mechanisms for maintaining GABAergic inhibition. However, it is interesting to note that the somewhat reduced α4 and elevated γ2 subunits in the δ subunit-deficient mouse (Korpi et al., 2002; Peng et al., 2002) do not form a sufficient number of extrasynaptic receptors with high affinity to produce a sizeable tonic conductance (Stell et al., 2003). Yet, as observed in the present study in DOCK10-Cre mice and in our previous study in C57BL/6 mice (Zhang et al., 2007), the pathological hyperexcitability during epileptogenesis initiates a process of much higher functional expression of the replacement subunits, as a tonic GABA conductance comparable to that seen in non-epileptic animals is present in the pilocarpine-treated GCs, albeit with a highly diminished neurosteroid sensitivity.

In the current study, the observed changes in labeling for the three subunits (δ, α4 and γ2) strongly suggest that, following the transfection-induced increase in δ subunit expression, the δ subunit competes successfully with the γ2 subunit for partnership with the α4 subunit. This is consistent with similar subunit competition during normal development, with the δ and α4 subunits emerging as preferred partners in the forebrain (Sur et al., 1999) and δ and α6 (homolog of the α4 subunit) as major partners in the cerebellum (Brickley et al., 2001; Tretter et al., 2001). Interestingly, studies in heterologous cells have demonstrated that the presence of the δ subunit is sufficient to inhibit assembly of the α6 and γ2 subunits (Martenson et al., 2017). The current findings are consistent with a similar process in vivo, with the induced increase in the δ subunit potentially inhibiting the partnership of α4 and γ2, thus allowing re-establishment of the α4/δ subunit partnership.

Provided the changes in α4 and γ2 subunit labeling in the current study lead to altered GABA_A_R subunit composition, as suggested, the function and pharmacology of the receptors could be altered. In numerous conditions in which excitability is increased, including epilepsy and alcohol-withdrawal, α4βγ2 GABA_A_Rs appear to be increased at the expense of the normally abundant α4βδ receptors (Cagetti et al., 2003; Olsen et al., 2005; Rajasekaran et al., 2010). Although these receptors may be capable of mediating tonic inhibition, they show reduced neurosteroid modulation (Brown et al., 2002; Rajasekaran et al., 2010; Zhang et al., 2007) and thus are unable to respond effectively to hormonal and stress-related changes as well as the periodic increases in excitability that characterize epilepsy (Belelli et al., 2002; Maguire et al., 2005; Stell et al., 2003). In addition, recombinant α4βγ2 receptors activated more slowly when exposed to low GABA concentrations and responded less effectively during repetitive firing (Lagrange et al., 2007). Thus, the combined changes in δ, α4 and γ2 expression that are found in the current study could contribute to normalization of subunit composition in some GABA_A_Rs and potentially lead to more effective inhibitory control.

Our electrophysiological findings demonstrate that the study of stimulus-response curves of fEPSPs, in the presence of GABA concentrations (5 μM) that activate the extrasynaptic GABA_A_Rs, is a sensitive measure of the activation of tonic inhibitory mechanisms in a large population of neurons. We have previously demonstrated that the loss of δ subunit-containing GABA_A_Rs in the pilocarpine model of temporal lobe epilepsy (TLE) (Peng et al., 2004) and during pregnancy (Maguire et al., 2009) increases network excitability in the dentate gyrus and cerebral cortex, respectively. The synaptic stimulus-response curves in these two studies have also shown the decreased sensitivity to neurosteroids when the expression of δ GABA_A_R subunits is downregulated. Our present findings indicate that restoring the expression of δ subunits in a TLE mouse model by unilateral viral transfection lowered neuronal excitability and reinstated the sensitivity of the fEPSPs to the neurosteroid THDOC. Our results are consistent with the idea that δ GABA_A_R subunit expression is a major factor in controlling the dendritic synaptic excitability of dentate gyrus GCs. The in vitro electrophysiological results provide strong proof of principle that selective transfection of δ subunits in GCs in TLE can decrease network excitability of the dentate gyrus. The effects of such increases in δ subunit expression on spontaneous seizures remain to be determined and will probably require increasing expression bilaterally and over the entire longitudinal extent in an epilepsy model. Future studies will have to determine the ideal timing of restoring δ subunits in GCs to reduce the severity of epilepsy, as well as finding potential means of preventing their reduced expression to abate epileptogenesis.

The current findings support the importance of δ subunit-containing receptors in controlling neuronal excitability in epilepsy, particularly in regions with high levels of δ subunit expression, such as the dentate gyrus and cerebellar cortex (Lee and Maguire, 2014; Rudolph et al., 2020). Deficits in δ subunit levels or surface expression have been found in several models of epilepsy and traumatic brain injury (Boychuk et al., 2016; Joshi et al., 2017; Lee et al., 2021; Parga Becerra et al., 2021; Peng et al., 2004; Rajasekaran et al., 2010; Schwarzer et al., 1997). (But also see Brooks-Kayal et al., 1998). In such forms of epilepsy, and other conditions with increased network excitability, such as fragile X syndrome (Martin et al., 2014; Zhang et al., 2017), enhancing δ subunit-mediated tonic inhibition could prove beneficial. Interestingly, despite the similar levels of tonic GABA currents measured at the level of dentate GCs before and after epilepsy (Zhang et al., 2007; and present study), there is a greater network excitability in the dentate of the epileptic animals (Peng et al., 2004). This result may potentially stem from the lack of neurosteroid sensitivity of the receptors that compensated for the loss of tonic conductance mediated by δ subunit-containing GABA_A_Rs, and may indicate that endogenous neurosteroids are present in the dentate gyrus, particularly during bouts of high excitability, that further dampen the excessive stimulation of GCs.

Despite the consistently observed decrease in δ subunit expression or surface localization in animal models, a recent study described an increase in δ subunit mRNA and immunolabeling in the dentate gyrus of patients with TLE (Sperk et al., 2021). While these findings could represent species differences in δ subunit responses in epilepsy, other factors could be considered. It is unknown whether surface expression of the δ subunit was also increased in the TLE specimens or if the subunit remained sequestered in the cytoplasm where it would be nonfunctional. Also, considering recent findings that in vivo administration of gabapentin can increase surface expression of δ subunit-containing receptors in the hippocampus (Yu et al., 2019), the potential effects of this and other antiepileptic drugs become important to consider in detail. The inherent difficulties in quantitative analyses and comparisons of immunolabeling in autopsy (control) and surgical specimens, with notably different intervals prior to fixation, remain an issue, as discussed by the investigators. The increased expression of the δ and α5 subunits was considered an endogenous anticonvulsant mechanism in TLE (Sperk et al., 2021) and, as such, could be sites for further enhancement of tonic inhibition, through activation of the δ- or α5-containing receptors with appropriate pharmacological agents.

The present findings support a search for new ways to increase δ subunit expression in neurons in which the δ subunit protein is decreased. Importantly, the findings suggest that some of the multiple GABA_A_R subunit alterations that are often found in disease models may be interrelated and, by identifying and modifying a critical subunit, related subunits may also be altered, presumably leading to GABA_A_Rs with a more normal subunit composition. While substantial progress has been made in assembling recombinant GABA_A_Rs with specific subunit compositions in cell lines, coordinated changes in GABA_A_R subunits in response to transfection of a single subunit in vivo have not been described previously in a disease process, even though such changes may have occurred.

Similar plasticity of GABA_A_R subunits has been observed in genetically-engineered mice that are deficient in a single GABA_A_R subunit. In such animals, other subunits often change their expression in the same brain region and cell type in which the subunit is lacking (Korpi et al., 2002; Kralic et al., 2006; Nusser et al., 1999; Peng et al., 2002; Tretter et al., 2001). However, these changes occur during the course of development, and, previously, it was uncertain whether such coordinated changes would occur in mature animals after the onset of a disease process.

The current results also call attention to the multiple processes that are required for the formation of functional GABA_A_Rs beyond increasing expression of a deficient GABA_A_R subunit. While the progression from δ subunit transfection to surface localization appears successful in the current study, it is unlikely to be complete, and each step in the process represents a site for additional interventions that could enhance normal GABA_A_R function.

Importantly, the GABA_A_Rs that assembled following δ subunit transfection reduced excitability of dentate GCs, and they were once again responsive to neurosteroid modulation, consistent with the functional characteristics of δ subunit-containing receptors. Together, these findings demonstrate that modifying a single step in the process of GABA_A_R formation in vivo can lead to broader changes in subunit expression and function in a disease model. The well-recognized plasticity of GABA_A_Rs and their subunit partnerships could thus serve as a framework for the development of new treatment approaches aimed at correcting basic GABA_A_R subunit deficits.
